# Heat shock factor 1 promotes proliferation and chemoresistance in diffuse large B-cell lymphoma by enhancing the cell cycle and DNA repair

**DOI:** 10.1038/s41419-025-07843-2

**Published:** 2025-07-17

**Authors:** Yu Fang, Liang Cheng, Ming Huang, Yi Cao, Qihua Zou, Jun Cai, Yuchen Zhang, Yi Xia, Huiqiang Huang, Xu Chen, Qingqing Cai

**Affiliations:** 1 Department of Oncology, Sun Yat-Sen Memorial Hospital, Sun Yat-Sen University, Guangzhou, Guangdong China; 2 Department of Urology, Sun Yat-sen Memorial Hospital, Sun Yat-sen University, Guangzhou, Guangdong China; 3 Guangdong Provincial Key Laboratory of Malignant Tumor Epigenetics and Gene Regulation, Department of Urology, Sun Yat-sen Memorial Hospital, Sun Yat-Sen University, Guangzhou, Guangdong China; 4 Guangdong Provincial Clinical Research Center for Urological Diseases, Department of Urology, Sun Yat-sen Memorial Hospital, Sun Yat-sen University, Guangzhou, Guangdong China; 5 State Key Laboratory of Oncology in South China, Collaborative Innovation Center for Cancer Medicine, Sun Yat-sen University Cancer Center, Guangzhou, Guangdong China; 6 Department of Medical Oncology, Sun Yat-sen University Cancer Center, Guangzhou, Guangdong, China

**Keywords:** B-cell lymphoma, Chemotherapy

## Abstract

Diffuse large B-cell lymphoma (DLBCL) is commonly treated with R-CHOP (rituximab, cyclophosphamide, doxorubicin, vincristine, and prednisone), but up to 40% of patients have refractory or relapsing disease and show unsatisfactory responses to salvage treatment. Heat shock factor 1 (HSF1) regulates the transcription of a group of oncogenes, promoting chemoresistance and representing a promising therapeutic target. However, the role and mechanism of HSF1 in DLBCL remain unknown. In this study, we discovered that the overexpression of HSF1 was correlated with unfavorable treatment response and poor prognosis in patients with DLBCL. Inhibition of HSF1 via shRNA or DTHIB, a pharmacological inhibitor of HSF1, inhibited cell proliferation and increased chemosensitivity to vincristine and doxorubicin both in vitro and in vivo. Mechanistically, we revealed that genes related to the cell cycle, DNA repair, and p53 signalling pathways, including CCNB1, CCNE2, E2F2, and XRCC2, were directly regulated by HSF1 in a protein arginine methyltransferase 5 (PRMT5) -dependent manner. These findings demonstrated that the significant transcriptional regulator HSF1 promoted cell proliferation and chemoresistance in DLBCL. Targeting HSF1 may serve as a promising therapeutic strategy that enhances the antitumor effects of chemotherapy in DLBCL.

## Introduction

Diffuse large B-cell lymphoma (DLBCL) is the most prevalent type of lymphoma [1]. The integration of rituximab, an anti-CD20 monoclonal antibody, with the CHOP regimen (cyclophosphamide, doxorubicin, vincristine, and prednisone [R-CHOP]) has significantly improved patient outcomes [2, 3]. While the majority of patients can be cured with R-CHOP, ~15% of patients exhibit refractory disease, and 20–25% experience relapse after an initial response [4, 5]. Numerous strategies have been tested in randomized trials to increase the efficacy of R-CHOP, including intensifying chemotherapy [6–8] or rituximab [9], using second-generation anti-CD20 monoclonal antibodies [10], adding maintenance therapy [11, 12], or incorporating novel agents [13, 14]; however, these trials have not resulted in significant improvements [4, 15]. Recently, the R-CHP regimen combined with the anti-CD79b antibody‒drug conjugate Polatuzumab Vedotin demonstrated superior progression-free survival (PFS) compared with R-CHOP in the POLARIX phase III trial. However, there were no significant differences in overall response rates, including complete response (CR) and partial response (PR) rates, or in overall survival (OS) [16]. Therefore, identifying novel prognostic indicators and therapeutic targets for DLBCL is clinically valuable for enhancing treatment efficacy and survival outcomes.

Heat shock transcription factors (HSFs) and heat shock proteins (HSPs) were originally identified for their role in maintaining proteome stability under environmental stress [17]. These proteins have also been shown to be overexpressed in many cancers, including bladder cancer, breast cancer, and intrahepatic cholangiocarcinoma, and are exploited by cancer cells to promote growth, survival, and metastasis [18, 19]. In DLBCL cells, HSP90 has been shown to interact with BCL6, stabilizing both BCL6 mRNA and protein and forming complexes with BCL6 at its target promoters. HSP90 inhibitors induce cell death in DLBCL, a process dependent on the transcriptional repression of BCL6 [20]. These findings suggest that HSPs may play a crucial role in the progression of DLBCL.

Heat shock factor 1 (HSF1) is a key regulator among HSFs [21]. Recent studies indicate that HSF1 also plays a compelling pro-oncogenic role by directing transcriptional programs that regulate oncogene expression instead of HSPs [22, 23]. HSF1 is involved in cell survival, proliferation, metabolism, and protein synthesis in various malignancies [22–24]. Array-based comparative genomic hybridization analysis revealed recurrent gains of chromosomal segments, including HSF1, in newly diagnosed canine DLBCL, suggesting a potential link between HSF1 expression and DLBCL onset [25]. However, whether and how HSF1 is involved in DLBCL, especially in chemoresistance, remain largely unknown.

In this study, we first revealed the clinicopathological relevance and roles of HSF1 in DLBCL. We then verified the pharmacological impact of Direct Targeted HSF1 Inhibitor (DTHIB) in enhancing cell proliferation and chemosensitivity in DLBCL. Finally, we elucidated the mechanisms by which HSF1 plays an oncogenic role through the transcriptional activation of target genes via interactions with PRMT5. The aim of this study was to facilitate the transition from in vitro and in vivo findings to preclinical and clinical trials for DLBCL treatment.

## Materials and methods

### Clinical samples

This study retrospectively collected clinical and survival data from 196 patients pathologically diagnosed with DLBCL at Sun Yat-sen University Cancer Center (SYSUCC) between January 2006 and December 2016. All included patients received first-line R-CHOP treatment. Additionally, formalin-fixed, paraffin-embedded (FFPE) primary DLBCL specimens from these patients were obtained. The project was approved by the institutional review board of SYSUCC (B2020-034). All specimens were acquired with written informed consent. The basic clinicopathological information is presented in Table S1.

### GEO data mining

Gene expression and clinical data from DLBCL patients were obtained from two Gene Expression Omnibus (GEO) datasets (GSE10846 and GSE117556). Patients with R-CHOP as first-line treatment, complete clinicopathological data and follow-up information were included in the Student’s *t* test and Kaplan‒Meier analysis. The basic clinicopathological features of the patients are listed in Table S2.

### Immunohistochemistry (IHC) and scoring

IHC staining was performed on FFPE tissue as previously described [26]. The specimens were first dewaxed and rehydrated and then incubated with protease K at 37 °C for 15 min to retrieve antigens. Endogenous peroxidase activity was blocked by treating the specimens with a 3% H_2_O_2_ solution for 10 min at 25 °C. Next, the specimens were incubated with primary antibodies overnight at 4 °C. The antibodies used to assess protein expression in the tissue samples are detailed in Table S3. After three washes with PBS, the specimens were incubated with biotinylated secondary antibodies for 1 h at 25 °C, after which color development was performed via DAB solutions (ZSGB-BIO, Beijing, China). Finally, the tissues were counterstained with hematoxylin.

IHC analyses were performed as previously described [26]. Two pathologists independently quantified HSF1 and PRMT5 expression in the specimens via a staining scoring system. Briefly, the staining intensity was rated as negative (0), weak (1), medium (2), or strong (3) (Fig. S1). A composite score ranging from 0 to 300 was calculated by multiplying the staining intensity by the percentage of stained cells. Cases were classified as low (score < 90) or high (score ≥ 90) expression levels of HSF1 or PRMT5. Images were captured with a Nikon ECLIPSE Ti microscope system (Tokyo, Japan) and analysed via Nikon software.

### Cell culture

The human DLBCL cell lines SU-DHL-2, SU-DHL-4, and SU-DHL-6 were purchased from BeNa Culture Collection (BNCC, Beijing, China). These cell lines were cultured in RPMI 1640 medium (Gibco, Carlsbad, California, USA) supplemented with 10% fetal bovine serum and 1% penicillin‒streptomycin at 37 °C with 5% CO_2_. They were tested negative for mycoplasma contamination and showed no misidentification or contamination with other cell lines after short tandem repeat (STR) authentication (IGE Biotechnology, Guangzhou, Guangdong, China).

### Stable HSF1-knockdown cell lines

The pLKO.1 TRC cloning vector was used to create short hairpin RNAs (shRNAs) targeting HSF1, PRMT5, or a negative control. The sequences for all the shRNAs are provided in Table S4. Lentivirus production and infection followed the procedures outlined in our previous study [26]. The plasmids were mixed with X-tremeGENE (Invitrogen, Carlsbad, CA, USA) and incubated at 25 °C for 20 min. The mixture was then added to the cells and incubated for 24–48 h. To produce lentiviruses, HEK-293T cells were transfected with PMD2. G (IGE), psPAX2 (IGE), and stably silenced vectors via X-tremeGENE. After a 48-h incubation period, the lentiviruses were collected, filtered, and concentrated. The target cells were then infected with the viruses in the presence of polybrene (IGE) and subsequently selected with puromycin.

### Real-time quantitative PCR (qRT‒PCR)

Total RNA was extracted from cells via TRIzol Reagent (Takara, Kusatsu, Shiga, Japan) according to the manufacturer’s instructions. Reverse transcription was carried out with the PrimerScript RT‒PCR Kit (Takara). Quantitative RT‒PCR was performed via a SYBR Green PCR kit (Vazyme, Nanjing, Jiangsu, China) on a LightCycler 480 system. The mRNA levels were quantified via the 2^-ΔΔCt^ method, with GAPDH serving as the internal control. The primer sequences are listed in Table S5.

### Western blotting assay and antibodies

Western blotting was performed following the methods described in our previous study [27]. The primary antibodies targeting HSF1, PRMT5, Cyclin B1, Cyclin E2, XRCC2, E2F2, MCM2, PCNA, p21, CDK2, Bcl-2, Caspase-3, Cleaved Caspase-3, PARP, Cleaved PARP and GAPDH are listed in Table S3. After incubation with primary antibodies, the membranes were incubated with the corresponding secondary antibodies. The protein bands were detected using enhanced chemiluminescence.

### Cell proliferation assays

Cell viability was measured using a Cell Counting Kit-8 (CCK8) assay. Briefly, 1 × 10^5^ cells were planted in 96-well culture plates and incubated with 20 μL of CCK8 reagent (APExBIO, Houston, Texas, USA) for 2 h. The formazan absorbance was then measured at 450 nm.

The cell cycle analysis was performed using the Cell Cycle Detection Kit (KGA512, KeyGEN, Nanjing, Jiangsu, China). DLBCL cells were collected, fixed with 70% cold ethanol at 4 °C overnight, and subsequently stained with PBS containing 50 μg/mL propidium iodide (PI) and 100 μg/mL RNase A for 30 minutes. Data were acquired via a FACS Vantage flow cytometer (BD Biosciences, San Jose, California, USA) and analysed with FlowJo software.

### Apoptosis analysis

Apoptosis analysis was carried out using the Annexin V-FITC/PI Apoptosis Detection Kit (KGA107, KeyGEN). DLBCL cells were harvested and stained with FITC-annexin V and PI, followed by flow cytometry analysis (BD Biosciences). Additionally, the TUNEL assay was performed using the In Situ Cell Death Detection Kit (Vazyme, Nanjing, Jiangsu, China), as described in previous studies [28].

### Chemosensitivity assay and drug synergistic effect calculations

DLBCL cells were exposed to various concentrations of DTHIB (Selleck Chemicals, Shanghai, China; 0, 1, 2, 4, 6, 8, or 12 μM), vincristine (Selleck; 0, 0.1, 0.2, 0.3, 0.4, 0.5, 1, or 2 nM), or doxorubicin (Selleck; 0, 0.03, 0.06, 0.125, 0.25, 0.5, or 1 μM) for 48 h. Cell viability was assessed using the CCK8 assay. IC_50_ values and dose‒response curves were calculated using GraphPad Prism 8. To evaluate the synergistic effects of DTHIB with vincristine or doxorubicin, DLBCL cells were treated with each drug individually or in combination, and viability was measured by CCK8 assay. The combination index (CI) was determined with CalcuSyn software (Biosoft), with a CI < 1.0 indicating a synergistic effect [29].

### RNA sequencing analysis

Total RNA was extracted from SU-DHL-2 or SU-DHL-6 cells treated with DMSO or DTHIB (8 μM) for 36 h using TRIzol reagent (Invitrogen). Library construction and sequencing were performed by Annoroad Gene Technology (Beijing, China). Sequencing was carried out on an Illumina NovaSeq 6000 platform, which produced 100 bp paired-end reads. All primary data in RNA sequencing (RNA-seq) analysis have been uploaded to the Gene Expression Omnibus (GSE295838).

### Chromatin immunoprecipitation (ChIP)

ChIP assays were performed following the manufacturer’s protocol (Thermo, Waltham, Massachusetts, USA) as previously described [26]. Briefly, after transfecting cells with HSF1 or PRMT5 shRNA or control shRNA, or treating them with DTHIB or DMSO for 48 h, DLBCL cells were crosslinked with 1% formaldehyde and then neutralized with glycine. The cells were lysed, sonicated, and incubated with anti-HSF1, anti-RNA polymerase II antibodies or with the negative control, IgG (Table S3) at 4 °C overnight. The chromatin‒antibody complexes were subsequently incubated with magnetic beads on ice for 2 h and collected. The beads were washed three times, and the DNA was purified using phenol-chloroform extraction followed by ethanol precipitation. The primers used for ChIP‒qPCR are listed in Table S6.

### Co-immunoprecipitation (Co-IP) and mass spectrometry (MS) analysis

Co-IP was carried out as described in our previous studies [26]. We examined the interaction between endogenous HSF1 and PRMT5 in wild-type SU-DHL-2, SU-DHL-4, and SU-DHL-6 cells. Briefly, nuclear extracts from the cells were incubated with anti-HSF1, anti-PRMT5, or control IgG (Table S3) at 4 °C overnight, followed by treatment with A/G magnetic beads at 25 °C for 2 h. Immunoreactive proteins in the lysates were analysed via western blotting. MS analysis was conducted at the Bioinformatics and Omics Center of Sun Yat-Sen Memorial Hospital.

### Xenograft tumor studies

All animal procedures in this study were approved by the Sun Yat-sen University Institutional Animal Care and Use Committee, with the approval number SYSU-IACUC-2021-000371. NOD-SCID mice, aged four weeks, were obtained from Beijing Vital River Laboratory Animal Technology, Beijing, China. For experiments involving HSF1 knockdown in combination with vincristine or doxorubicin, 1 × 10^7^ SU-DHL-6 cells stably transfected with either sh-control or sh-HSF1 were injected subcutaneously into the right flank of the randomized mice. After 10 days, the mice with sh-HSF1 or sh-control xenografts were randomly assigned to three treatment groups: vincristine (2 mg/kg), doxorubicin (2 mg/kg), or PBS. For the experiments combining DTHIB with vincristine or doxorubicin, 1 × 10^7^ wild-type SU-DHL-6 cells were injected subcutaneously into the right flank of the randomized mice, and the mice with xenografts were randomly divided into six groups: (1) Control group, which was treated with PBS every 3 days; (2) DTHIB group, which was treated with DTHIB at 5 mg/kg every 3 days; (3) Vincristine group, which was treated with vincristine at 2 mg/kg every 3 days; (4) Doxorubicin group, which was treated with doxorubicin at 2 mg/kg every 3 days; (5) Combination group 1, which was treated with DTHIB and vincristine every 3 days; and (6) Combination group 2, which was treated with DTHIB and doxorubicin every 3 days. Treatments were continued for three weeks. Tumor volumes were measured every 3 days, and tumors were surgically excised following the euthanasia of the mice. Finally, the tumors were fixed in 4% paraformaldehyde and embedded in paraffin. No statistical method was used to predefine the sample size for the mouse experiments; the sample size was determined based on prior experimental experience. During the experiments, the investigator was blinded to group allocation to minimize potential bias.

### Statistical analysis

All the statistical analyses were conducted using GraphPad Prism 8.0 software. For two-group comparisons, a two-tailed Student’s *t* test was used, whereas for multiple group comparisons, two-way ANOVA followed by Dunnett’s test was used. The correlations between HSF1 expression and clinicopathological variables were evaluated using Pearson’s chi-square test and the correlations between two variables were analyzed via Spearman’s correlation analysis. Survival rates were analysed with Kaplan‒Meier analysis and the log-rank test. Cox proportional hazard regression models were employed to calculate survival hazard ratios and 95% confidence intervals. All statistical tests were two-sided. Data from at least three independent experiments are presented as the means ± SDs, and differences were considered statistically significant at *p* < 0.05.

## Results

### High expression of HSF1 was associated with unfavorable treatment response and poor prognosis in DLBCL patients

**Fig. 1:**
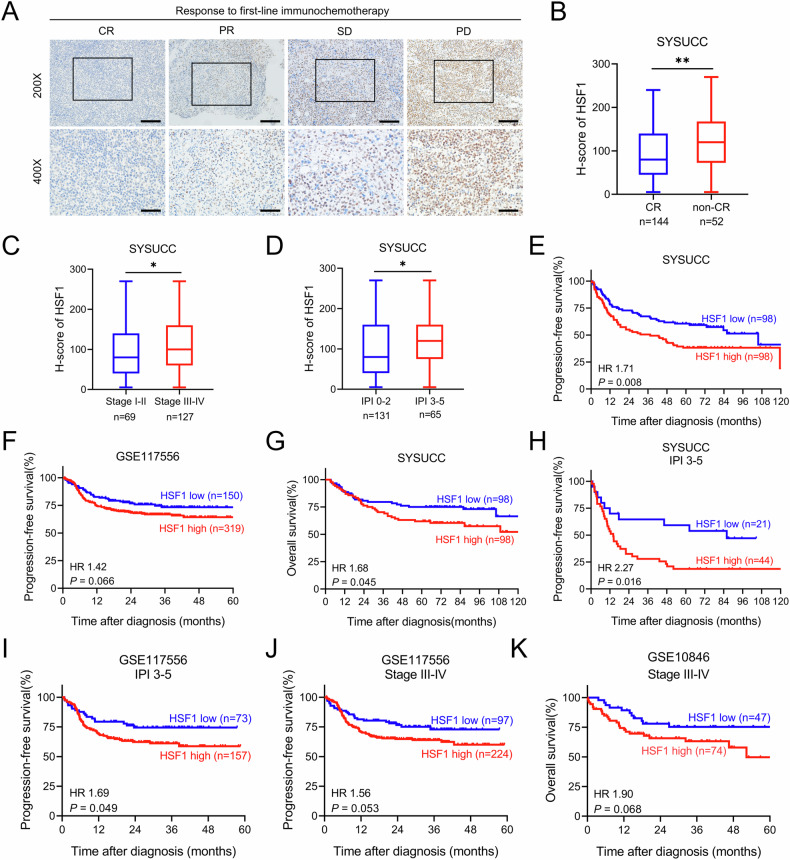
High HSF1 expression is correlated with poor therapeutic response and survival prognosis in DLBCL patient cohorts. **A** Representative IHC images of HSF1 in DLBCL patients with the best response of complete response (CR), partial response (PR), stable disease (SD) and progressive disease (PD) after first-line R-CHOP treatment. Scale bars in 200×: 100 μm; scale bars in 400×: 50 μm. **B** Comparison of HSF1 expression between patients who achieved CR and those who did not achieve CR after first-line R-CHOP treatment. **C** Comparison of HSF1 expression between patients with early-stage disease and those with advanced-stage disease. **D** The expression of HSF1 in patients with an IPI of 0–2 point or 3–5 point. **E**, **F** Kaplan‒Meier curves for progression-free survival of patients in the SYSUCC and GSE117556 cohorts. **G** Kaplan‒Meier curves for overall survival of patients in the SYSUCC cohort. **H**, **I** Kaplan‒Meier curves for progression-free survival of patients in the IPI 3–5 subgroups of the SYSUCC and GSE117556 cohorts. **J** Kaplan‒Meier curves for progression-free survival of patients in the advanced-stage subgroup of the GSE117556 cohort. **K** Kaplan‒Meier curves for overall survival of patients in the advanced-stage subgroup of the GSE10846 cohort. *p < 0.05, **p < 0.01.

To determine the clinical relevance of HSF1 in DLBCL, we analysed HSF1 expression in 196 archived FFPE DLBCL tissue samples from SYSUCC using immunohistochemistry. We assessed the association between HSF1 expression and the treatment response to first-line R-CHOP therapy. Our results revealed that HSF1 expression was significantly elevated in tissues from patients with progressive disease (PD) after first-line chemotherapy and decreased as the treatment response improved (Fig. 1A). Specifically, HSF1 levels were notably higher in patients who achieved non-CR than in those who achieved CR (Fig. 1B). Furthermore, HSF1 overexpression was significantly associated with unfavorable clinical features, including advanced stage and high International Prognostic Index (IPI) scores (Fig. 1C, D). Kaplan-Meier analysis revealed that high HSF1 expression was associated with shorter PFS in both the SYSUCC cohort and the GSE117556 cohort (Fig. 1E, F) and with decreased OS in the SYSUCC cohort (Fig. 1G). In a subgroup analysis of patients with adverse clinical profiles, such as those with an IPI score of 3–5, high HSF1 levels were linked to poor PFS in the SYSUCC and GSE117556 cohorts (Fig. 1H, I). For patients with advanced-stage disease, high HSF1 expression was similarly associated with worse PFS in the GSE117556 cohort (Fig. 1J) and with poorer OS in the GSE10846 cohort (Fig. 1K). Univariate Cox regression analysis confirmed that high HSF1 expression was significantly associated with an increased risk of disease progression and overall mortality (Tables S7-8). Overall, these findings indicate that HSF1 overexpression is associated with unfavorable treatment responses and poor prognosis, suggesting its potential as a clinical biomarker for DLBCL.

### HSF1 knockdown inhibited proliferation, and enhanced apoptosis and sensitivity to vincristine and doxorubicin in DLBCL in vitro

**Fig. 2:**
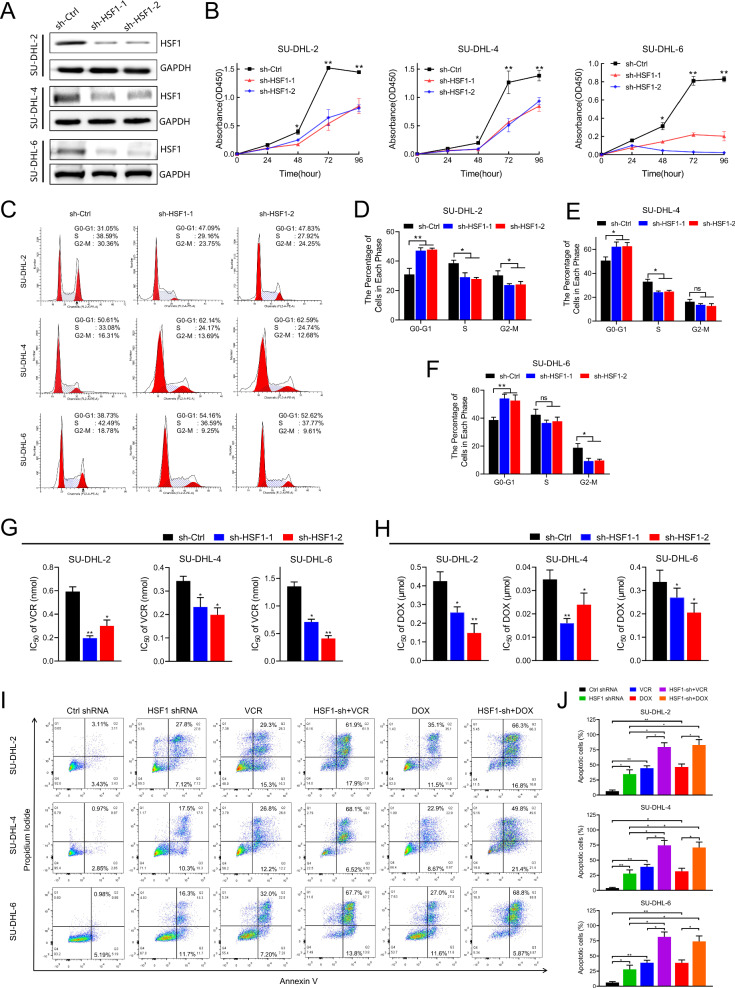
HSF1 knockdown inhibited proliferation, and enhanced apoptosis and sensitivity to vincristine and doxorubicin in DLBCL in vitro. **A** Western blotting analysis of HSF1 expression in HSF1-silenced cells and control cells. GAPDH was used as a control. **B** CCK8 assays of SU-DHL-2, SU-DHL-4 and SU-DHL-6 cells transfected with HSF1 or Ctrl shRNA. Representative images (**C**) and quantification (**D**–**F**) of cell cycle in SU-DHL-2, SU-DHL-4 and SU-DHL-6 cells transfected with HSF1 or Ctrl shRNA were analysed by flow cytometry. IC_50_ values of vincristine (**G**) and doxorubicin (**H**) in SU-DHL-2, SU-DHL-4 and SU-DHL-6 cells with HSF1 knockdown compared with those in the respective control cells. Images (**I**) and quantification (**J**) of cell apoptosis in DLBCL cells transfected with control or HSF1 shRNA and treated with vincristine or doxorubicin for 48 h. Statistical significance was evaluated by using a two-tailed *t* test or one-way ANOVA. The error bars indicate the standard deviations (SDs) of three independent experiments. *p < 0.05, **p < 0.01.

To investigate the function of HSF1 in DLBCL, we established HSF1-knockdown SU-DHL-2, SU-DHL-4, and SU-DHL-6 cell lines and confirmed the knockdown efficiency using western blotting (Fig. 2A). The CCK8 assay revealed a significant decrease in cell proliferation following HSF1 silencing (Fig. 2B). Additionally, flow cytometry analysis revealed that HSF1 knockdown increased the percentage of cells in the G0/G1 phase and decreased the percentage in the S phase (Fig. 2C–F). These results suggest that HSF1 promotes cell proliferation by regulating the G1/S phase transition.

We then investigated whether silencing HSF1 could increase the chemosensitivity of DLBCL cells to vincristine or doxorubicin. Interestingly, CCK8 assays demonstrated that HSF1 depletion significantly increased the sensitivity of DLBCL cells to both vincristine and doxorubicin. The IC_50_ values for vincristine and doxorubicin were markedly lower in HSF1-knockdown cells than in control cells (Fig. 2G, H and Fig. S2). Moreover, the Annexin V/PI apoptotic assay revealed that the proportion of apoptotic DLBCL cells transfected with HSF1 shRNA was greater than that transfected with control shRNA, especially after treatment with vincristine or doxorubicin (Fig. 2I, J). We further validated the expression of key cell cycle and apoptosis-related proteins in HSF1-knockdown DLBCL cells. Consistently, HSF1 knockdown led to upregulation of p21 and downregulation of CDK2 (Fig. S3). Additionally, the levels of BCL2, Caspase-3 and PARP decreased following HSF1 knockdown, with a corresponding significant accumulation of cleaved Caspase-3 and cleaved PARP. These findings indicate that HSF1 is a crucial regulator of both proliferation and chemoresistance in DLBCL cells.

### HSF1 knockdown inhibited tumorigenesis and increased chemosensitivity to vincristine and doxorubicin in DLBCL in vivo

**Fig. 3:**
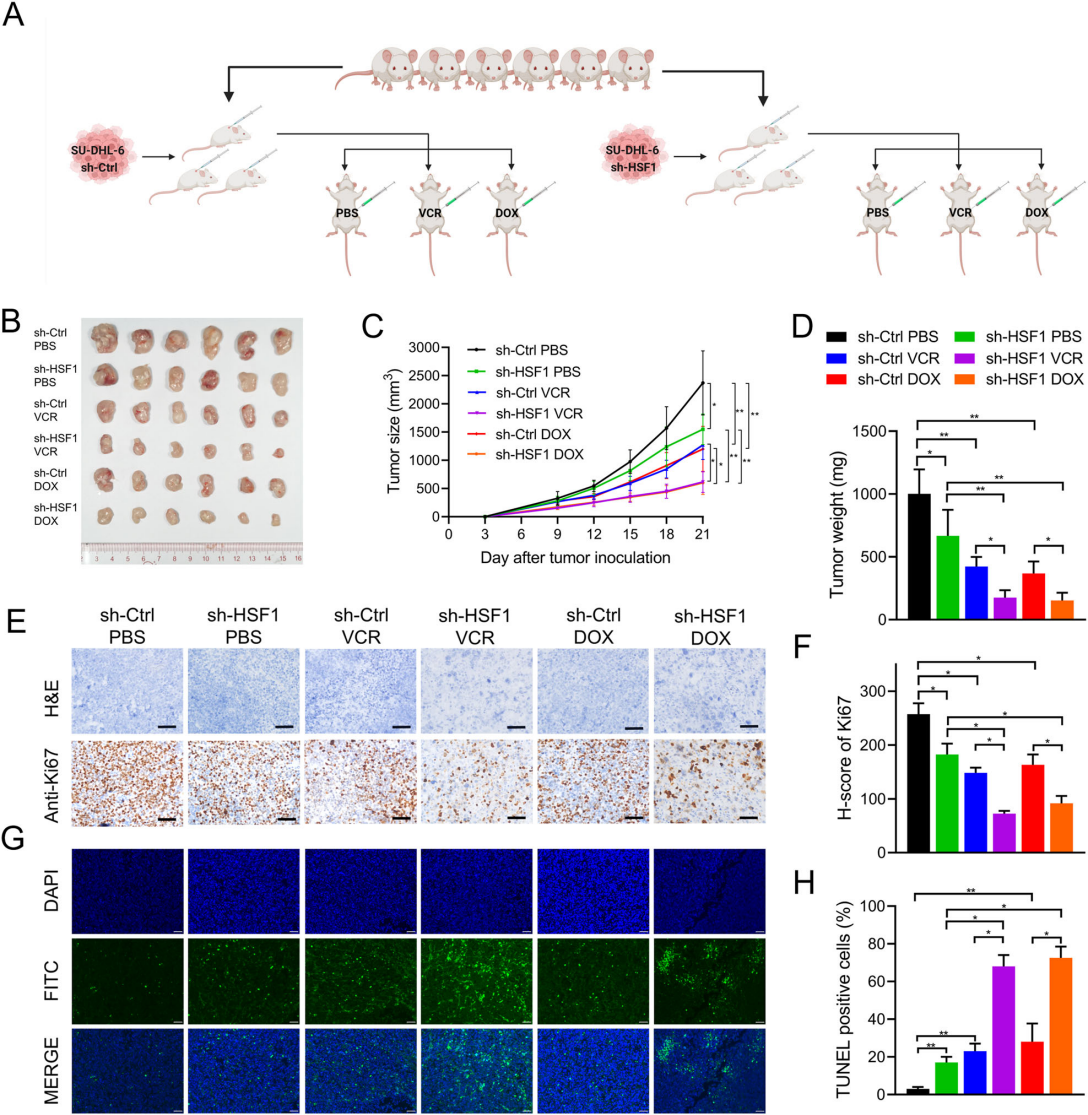
HSF1 knockdown inhibited tumorigenesis and increased chemosensitivity to vincristine and doxorubicin in DLBCL in vivo. **A** Schematic illustration of the establishment of the in vivo chemotherapy assay model. **B** Images of surgically dissected tumors in indicated groups. **C** The volume of tumors in indicated groups was measured every 3 days. The results are shown as the means ± SDs of the values (*n* = 6). **D** The weights of the tumors in indicated groups were measured after the tumors were surgically dissected. The results are shown as the means ± SDs (n = 6). Representative images (**E**) and H-scores (**F**) of Ki67 expression in tumors from indicated groups, as examined by IHC. Representative images of apoptosis in tumors (**G**) and the percentage of TUNEL-positive cells (**H**) in indicated groups. The scale bars in the IHC and TUNEL images represent 50 μm. *p < 0.05, **p < 0.01.

To further investigate the tumorigenesis-promoting and chemoresistance effects of HSF1 in vivo, we established subcutaneous xenograft mouse models (Fig. 3A). Stable HSF1 knockdown SU-DHL-6 cells (SU6-sh-HSF1) or control cells (SU6-sh-control) were injected subcutaneously into NOD-SCID mice. One week after inoculation, the mice bearing SU6-sh-HSF1 or SU6-sh-control xenografts were randomly assigned to treatment groups receiving PBS, vincristine, or doxorubicin, with tumors measured every three days. As shown in Fig. 3B–D, HSF1 knockdown resulted in slower tumor growth and lower tumor weights than those in the control group, with these effects being more pronounced in the vincristine and doxorubicin treatment groups than in the PBS group. Furthermore, the expression of the proliferation marker Ki67 was reduced, but the proportion of apoptotic cells was increased in tumors derived from HSF1-knockdown cells, particularly in the vincristine and doxorubicin treatment groups, compared with the respective control groups (Fig. 3E–H). Collectively, these data demonstrate that targeting HSF1 inhibits DLBCL cell growth and enhances sensitivity to vincristine and doxorubicin in vivo.

### The HSF1 inhibitor DTHIB suppressed proliferation and enhanced chemosensitivity in DLBCL cells

**Fig. 4:**
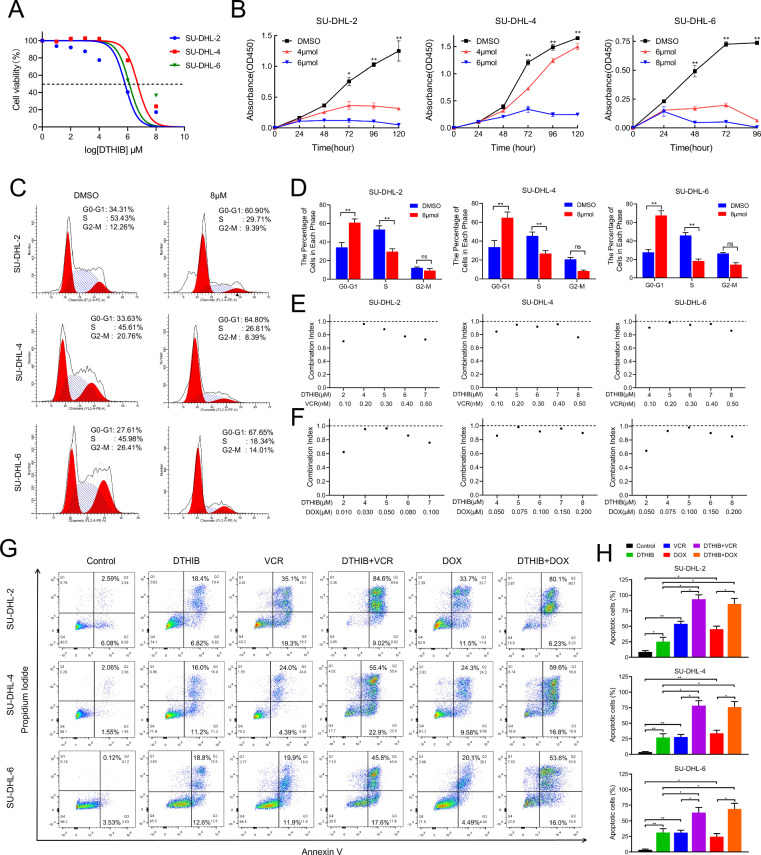
DTHIB suppressed proliferation and enhanced chemosensitivity in DLBCL cells. **A** Viability of SU-DHL-2, SU-DHL-4 and SU-DHL-6 cells under DTHIB treatment. Cells were treated with indicated concentrations of DTHIB for 48 h, and the viability was calculated via a CCK8 assay. Data are presented as means ± SDs of three independent experiments. **B** CCK8 assays of SU-DHL-2, SU-DHL-4 and SU-DHL-6 cells treated with DTHIB or DMSO at the indicated time points. Representative images (**C**) and quantification (**D**) of the cell cycle in SU-DHL-2, SU-DHL-4 and SU-DHL-6 cells treated with DTHIB or DMSO were analysed by flow cytometry analysis. Combination index (CI) of SU-DHL-2, SU-DHL-4 and SU-DHL-6 cells treated with a combination of DTHIB and vincristine (**E**) and a combination of DTHIB and doxorubicin (**F**). The CI was calculated via CalcuSyn software. A CI less than 1.0 indicates synergy between two drugs. Images (**G**) and quantification (**H**) of cell apoptosis in DLBCL cells treated with single agent or combination of DTHIB, vincristine and doxorubicin for 48 h. *p < 0.05, **p < 0.01.

To evaluate the translational potential of HSF1, we examined the effects of DTHIB, a pharmacological inhibitor of HSF1, in DLBCL cells. CCK8 assays revealed that DTHIB treatment significantly reduced DLBCL cell viability in a dose-dependent manner (Fig. 4A, B). Flow cytometry analysis further revealed that DTHIB induced G0/G1 phase arrest in DLBCL cells (Fig. 4C, D).

To determine whether the combination of DTHIB with vincristine or doxorubicin could synergistically inhibit the viability of DLBCL cells, we calculated the combination index (CI) values using the Chou‒Talalay median-effect principle [29]. Interestingly, DTHIB demonstrated a synergistic effect with both vincristine and doxorubicin in inhibiting the viability of DLBCL cells (Fig. 4E, F and Fig. S4). Moreover, DTHIB reinforced the effects of vincristine and doxorubicin on inducing cell apoptosis in DLBCL cells (Fig. 4G, H). Consistently, DTHIB treatment resulted in significant alterations in key proteins involved in cell cycle regulation and apoptosis (Fig. S5). These results suggest that DTHIB not only inhibits proliferation but also enhances the chemosensitivity of DLBCL cells in vitro.

### DTHIB inhibited tumorigenesis and enhanced the efficacy of vincristine and doxorubicin in DLBCL in vivo

**Fig. 5:**
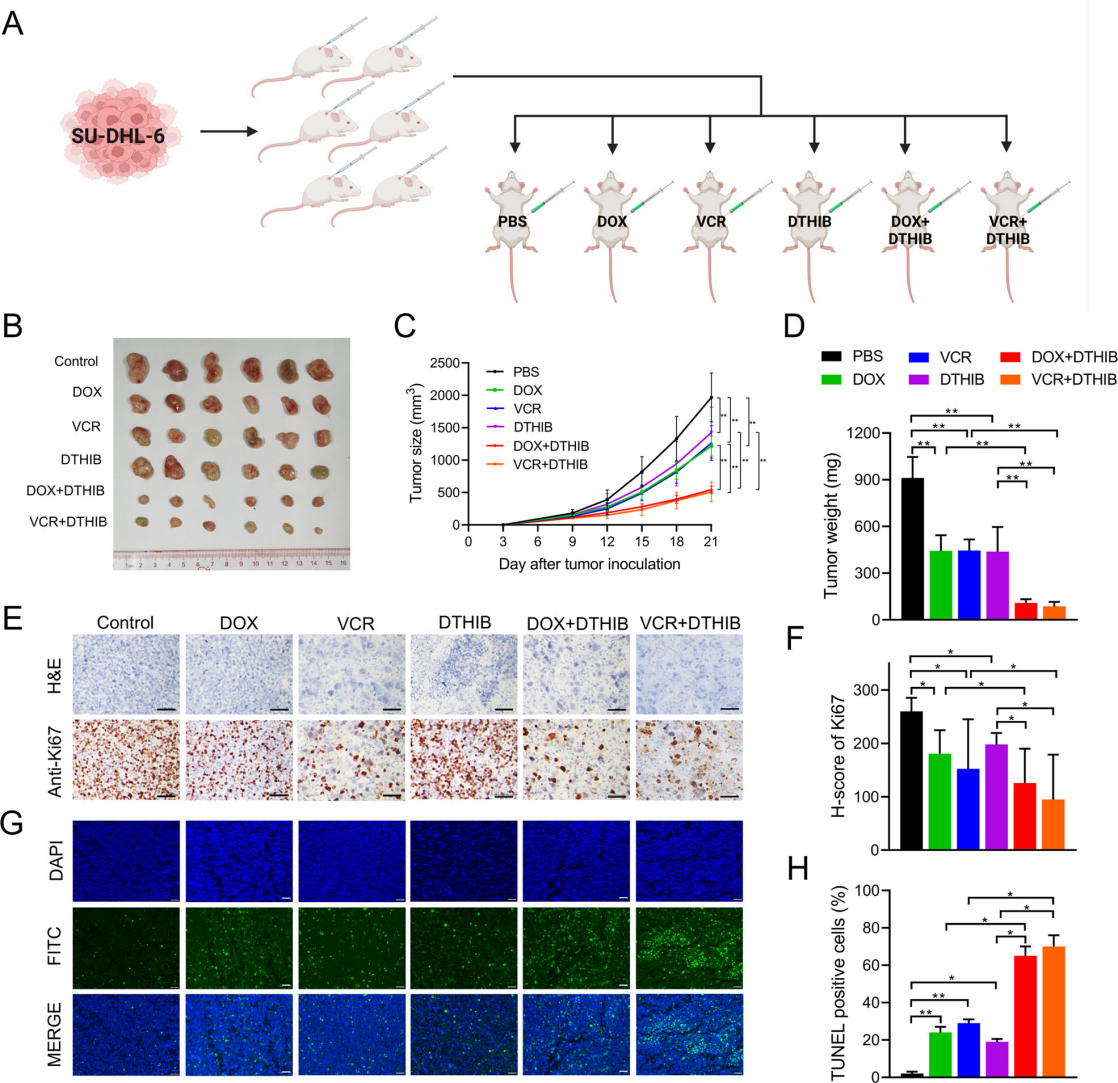
DTHIB inhibited tumorigenesis and enhanced the efficacy of vincristine and doxorubicin in DLBCL in vivo. **A** Schematic illustration of the establishment of the in vivo chemotherapy assay model. **B** Images of surgically dissected tumors in indicated groups. **C** The volume of tumors in indicated groups was measured every 3 days. **D** The weights of the tumors in indicated groups were measured after the tumors were surgically dissected. The results are presented as the means ± SDs (n = 6). **E**, **F** Representative images (**F**) and H-scores of Ki67 (**E**) in tumors from indicated groups. Representative images of apoptosis in tumors (**G**) and the percentage of TUNEL-positive cells (**H**) in indicated groups. The scale bars in the IHC and TUNEL images represent 50 μm. *p < 0.05, **p < 0.01.

To further evaluate the pharmacological effects of DTHIB in vivo, wild-type SU-DHL-6 cells were injected subcutaneously into NOD-SCID mice (Fig. 5A). One week postinjection, the mice were randomly assigned to six groups and treated every three days with PBS, DTHIB alone, vincristine alone, doxorubicin alone, DTHIB combined with vincristine, or DTHIB combined with doxorubicin. As shown in Fig. 5B–D, DTHIB treatment significantly increased the sensitivity of DLBCL to vincristine and doxorubicin chemotherapy, as evidenced by reduced tumor volume and weight. Notably, compared with treatment with vincristine or doxorubicin alone, the combination of DTHIB with either vincristine or doxorubicin also led to a reduction in Ki67 expression and an increase in the proportion of apoptotic cells (Fig. 5E–H). Additionally, histological analysis with HE staining revealed no alterations in the kidney, liver, lung, or heart tissues in the DTHIB-treated groups (Fig. S6). Compared with the use of vincristine or doxorubicin alone, combining DTHIB with either doxorubicin or vincristine did not result in increased toxicity. Collectively, these data demonstrate that DTHIB is a promising anti-tumor agent and that combining DTHIB with vincristine or doxorubicin could be a novel strategy for the treatment of DLBCL.

### The target genes of HSF1 and DTHIB in DLBCL

**Fig. 6:**
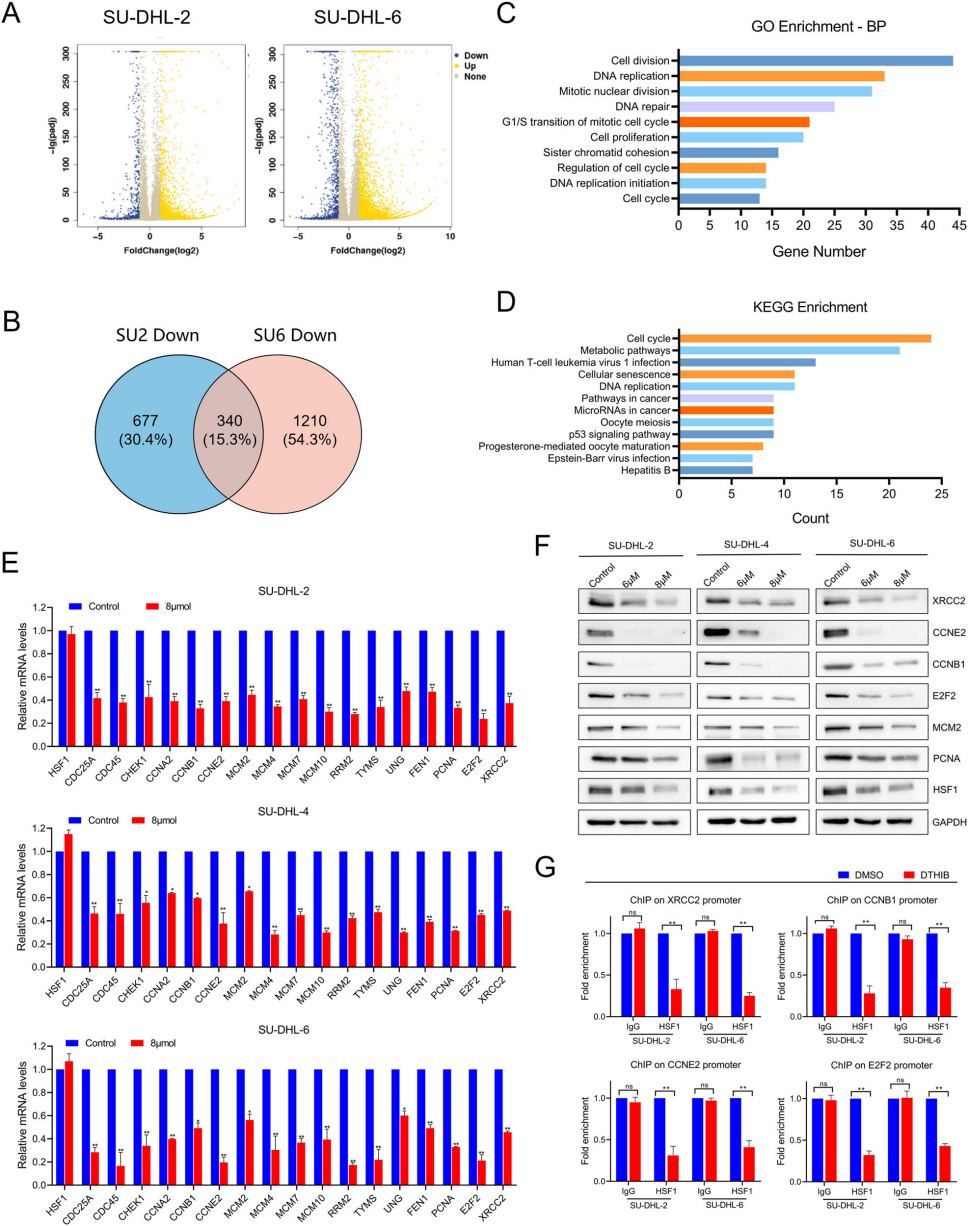
The target genes of HSF1 were identified in DLBCL cells. **A** Volcano plots displaying the gene expression changes in SU-DHL-2 (left) and SU-DHL-6 (right) regulated by DTHIB, compared with DMSO. Differential expression values are plotted against *p* values; yellow dots represent the upregulated genes, and blue dots represent downregulated genes. **B** Venn diagram showing the number of downregulated genes in SU-DHL-2 (left) and SU-DHL-6 (right) treated with DTHIB. A total of 677 genes were downregulated in SU-DHL-2, 1210 genes were downregulated in SU-DHL-6, and 340 genes were downregulated in both SU-DHL-2 and SU-DHL-6. **C** Gene Ontology (GO) analysis identifying the enrichment of biological processes. **D** Kyoto Encyclopedia of Genes and Genomes (KEGG) analysis identifying the enrichment of signaling pathways. Validation of candidate downregulated genes by qRT‒PCR (**E**) and Western blotting (**F**) in SU-DHL-2, SU-DHL-4 and SU-DHL-6. GAPDH was used as an internal control. **G** Chromatin immunoprecipitation (ChIP)-qPCR analysis of negative control IgG and HSF1 enrichment at the promoters of HSF1 target genes in SU-DHL-2 and SU-DHL-6 cells treated with DTHIB or DMSO. The values are normalized to the input values and are presented as the means ± SDs. *p < 0.05, **p < 0.01.

To further elucidate the mechanism of HSF1-mediated transcriptional regulation in DLBCL, we conducted transcriptomic sequencing on SU-DHL-2 and SU-DHL-6 cells treated with or without DTHIB (8 μM). We identified 340 genes that were downregulated in both DTHIB-treated SU-DHL-2 and SU-DHL-6 cell lines (Fig. 6A, B). Gene Ontology (GO) and Kyoto Encyclopedia of Genes and Genomes (KEGG) analyses revealed that these differentially expressed genes were involved mainly in cell cycle regulation, DNA replication, DNA repair, and the p53 signaling pathway (Fig. 6C, D). We then performed qRT‒PCR to validate the expression of target genes identified from the RNA-seq data. As shown in Fig. 6E, the expression of cell cycle-related genes (CCNB1, CCNA2, CCNE2, and E2F2), DNA repair-related genes (MCM2, XRCC2, FEN1, and UNG), and genes in the p53 signaling pathway (CHEK1 and RRM2) were significantly downregulated in DTHIB-treated cells. Similarly, these genes were also downregulated after HSF1 knockdown with two shRNAs (Fig. S7). Furthermore, western blotting analysis demonstrated that both DTHIB treatment and HSF1 knockdown reduced the protein expression levels of XRCC2, Cyclin E2, Cyclin B1, E2F2, MCM2, and PCNA (Fig. 6F and Fig. S8).

To investigate the regulatory effect of HSF1 on these genes, we performed chromatin immunoprecipitation (ChIP) assays on HSF1-knockdown and DTHIB-treated DLBCL cells, along with their respective controls. As depicted in Fig. 6G, the enrichment of HSF1 on the promoter regions of XRCC2, CCNB1, CCNE2, and E2F2 was lower in DTHIB-treated cells than in control cells. Similarly, HSF1 knockdown resulted in decreased HSF1 binding to the promoter regions of these target genes (Fig. S9). Furthermore, we analysed the promoter regions of the four target genes via the JASPAR database (http://jaspar.genereg.net) to predict potential HSF1 binding sites (Fig. S10). These findings demonstrate that HSF1 directly binds to the promoters of XRCC2, CCNB1, CCNE2, and E2F2 to regulate their transcription.

### HSF1 interacted with PRMT5 to promote tumorigenesis and enhance the chemosensitivity of DLBCL cells

**Fig. 7:**
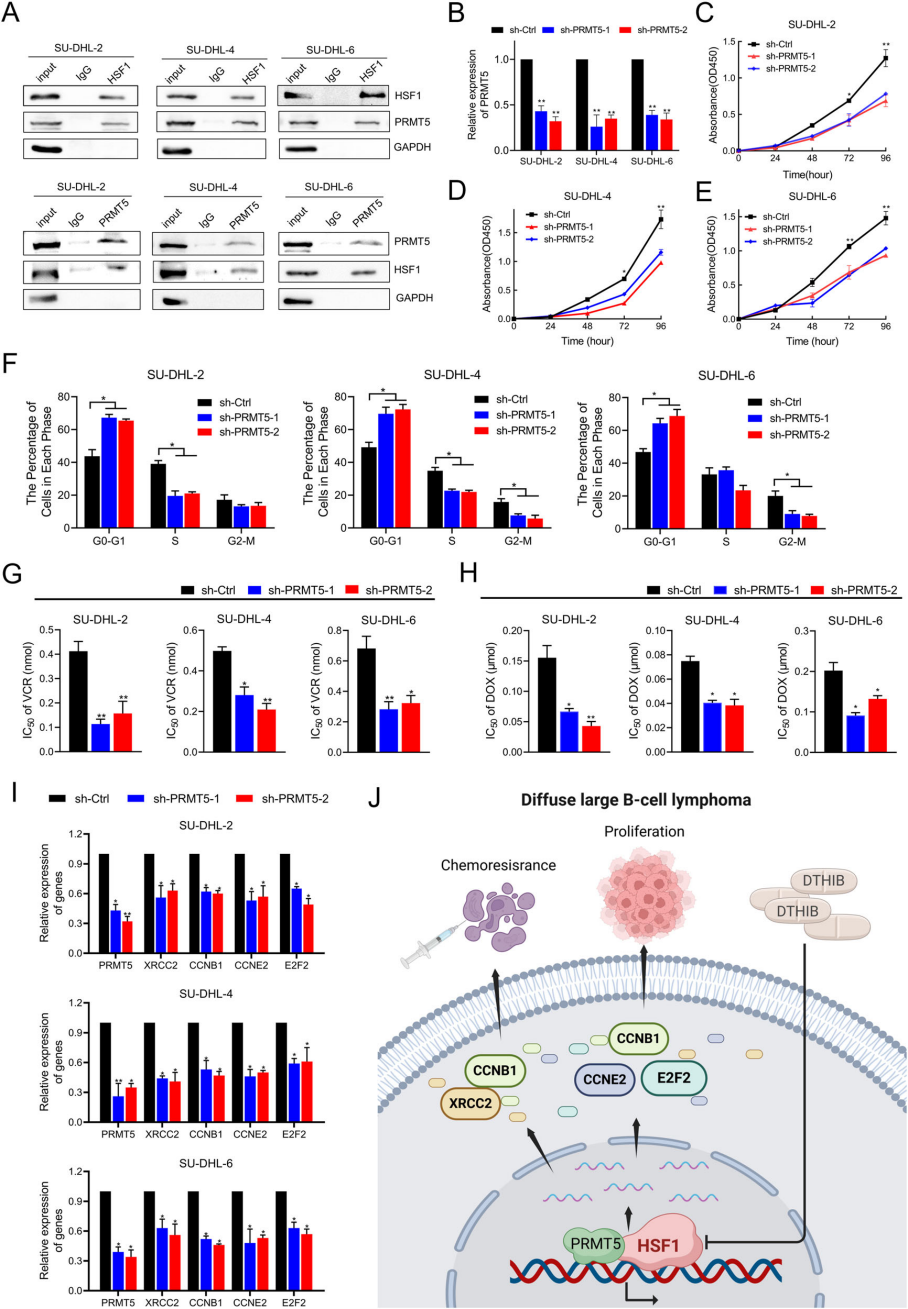
HSF1 regulated target gene expression by interacting with PRMT5. **A** Co-IP and Western blotting analysis showing the interaction between endogenous HSF1 and PRMT5. **B** qRT‒PCR analysis of PRMT5 expression in PRMT5-silenced cells and control cells. **C**–**E** CCK8 assays of SU-DHL-2, SU-DHL-4 and SU-DHL-6 cells transfected with PRMT5 or Ctrl shRNA. **F** Quantification of cell cycle in SU-DHL-2, SU-DHL-4 and SU-DHL-6 cells transfected with PRMT5 or Ctrl shRNA, analyzed by flow cytometry analysis. IC_50_ values of vincristine (**G**) and doxorubicin (**H**) in SU-DHL-2, SU-DHL-4 and SU-DHL-6 cells with PRMT5 knockdown, compared with those in respective control cells. **I** Validation of genes downregulated by qRT‒PCR in SU-DHL-2, SU-DHL-4 and SU-DHL-6 cells transfected with PRMT5 or Ctrl shRNA. **J** Illustrative model showing the underlying mechanism by which HSF1 promotes chemoresistance and cell proliferation in DLBCL via a PRMT5-dependent transcriptional program. The image was created via BioRender.com. Statistical significance was evaluated by using a two-tailed *t* test or one-way ANOVA. The error bars indicate the standard deviations of three independent experiments. *p < 0.05, **p < 0.01.

Given that HSF1 is a transcription factor that binds to specific proteins to regulate transcription [30], we conducted Co-IP and mass spectrometry (MS) assays in DLBCL cells. Among the HSF1-binding proteins identified by MS, PRMT5, a methyltransferase, has been recognized as a key regulator in B-cell lymphomas [31, 32] (Fig. S11). Further Co-IP and western blotting experiments confirmed the interaction between endogenous HSF1 and PRMT5 in wild-type SU-DHL-2, SU-DHL-4, and SU-DHL-6 cells (Fig. 7A). We subsequently evaluated the expression of HSF1 and PRMT5 by immunohistochemistry in 127 archived FFPE DLBCL tissue samples from the SYSUCC cohort (Fig. S12A). A positive correlation between HSF1 and PRMT5 expression was observed (Fig. S12B). Furthermore, Kaplan‒Meier survival analysis revealed that high PRMT5 expression was significantly associated with shorter PFS (Fig. S12C) and OS (Fig. S12D).

To determine whether PRMT5 has biological functions similar to those of HSF1 in DLBCL, we established PRMT5-knockdown SU-DHL-2, SU-DHL-4, and SU-DHL-6 cells and verified the knockdown efficiency using qRT‒PCR (Fig. 7B). CCK8 assays revealed that PRMT5 knockdown significantly reduced cell proliferation (Fig. 7C–E). Flow cytometry revealed that PRMT5 knockdown increased the percentage of DLBCL cells in the G0/G1 phase and decreased the percentage of DLBCL cells in the S phase (Fig. 7F and Fig. S13). Furthermore, PRMT5 knockdown increased sensitivity to vincristine and doxorubicin in vitro (Fig. 7G, H and Fig. S14). Additionally, PRMT5 knockdown inhibited the expression of XRCC2, CCNE2, CCNB1, and E2F2 (Fig. 7I). Intriguingly, PRMT5 knockdown did not affect HSF1 occupancy at the promoter regions of these target genes (Fig. S15). In conclusion, HSF1 interacts with PRMT5 to promote tumorigenesis and enhance the chemosensitivity of DLBCL cells.

## Discussion

HSF1 has been extensively studied and is recognized as essential for carcinogenesis and tumor growth. However, whether HSF1 is involved in the malignant progression of DLBCL and the underlying mechanisms remain unclear. In this study, we found that HSF1 overexpression was associated with poor treatment response and unfavorable prognosis in patients with DLBCL. Targeting HSF1 with shRNA or DTHIB significantly suppressed cell proliferation and enhanced chemosensitivity to vincristine and doxorubicin both in vitro and in vivo. HSF1 was shown to directly regulate key genes involved in the cell cycle, DNA repair, and p53 signaling pathways, including CCNB1, CCNE2, E2F2, and XRCC2, in a PRMT5-dependent manner (Fig. 7J). Collectively, these findings improve our understanding of chemoresistance and offer potential strategies for predicting prognosis and optimizing precision therapies in DLBCL.

Despite the significant improvement in the prognosis of DLBCL patients with the introduction of CD20 monoclonal antibody therapy, ~40% of patients still experience primary refractory disease or relapse after achieving complete remission. Even with salvage chemotherapy and autologous stem cell transplantation (ASCT), nearly 80% of these patients eventually succumb to lymphoma [3, 33]. Therefore, it is crucial to identify new prognostic biomarkers and therapeutic targets for DLBCL patients. In this study, we identified a significant positive correlation between elevated HSF1 expression and resistance to first-line treatment, along with poorer survival outcomes in DLBCL patients, particularly among those with adverse clinical characteristics, such as advanced-stage disease and high IPI scores. These results highlight the role of HSF1 in promoting DLBCL and its potential as a marker for predicting malignant behavior in DLBCL patients.

Previous studies have shown that HSF1 plays an oncogenic role in promoting proliferation and chemoresistance in various malignancies [34]. Consistent with these findings, our study demonstrated that HSF1 knockdown suppressed DLBCL cell proliferation by modulating cell cycle progression and significantly enhanced their sensitivity to vincristine and doxorubicin both in vitro and in vivo. While prior research on HSF1-mediated chemoresistance has focused largely on platinum agents [35–37], evidence on HSF1-related resistance to doxorubicin and vincristine remains limited. These results suggest that targeting HSF1 could be a promising therapeutic strategy when combined with first-line R-CHOP treatment for DLBCL patients. In recent years, checkpoint inhibitors such as anti-PD-1 antibodies have been widely employed in the treatment of various solid tumors, significantly improving patient outcomes. Previous studies have shown that phosphorylation of HSF1 at threonine 120 promotes its binding to the PD-L1 promoter, thereby increasing PD-L1 expression in breast cancer [38]. Similarly, in hepatocellular carcinoma, HSF1 has been reported to upregulate PD-L1 expression by inducing APOJ expression and activating the STAT3 signaling pathway [39]. These findings suggest that HSF1 may contribute to resistance to anti-PD-1 therapy and that targeting HSF1 could improve the efficacy of immunotherapy.

The target genes of HSF1 vary across different tumor types, and the specific targets of HSF1 in DLBCL remain unclear. This study demonstrated that HSF1 directly regulates essential genes related to the cell cycle, DNA damage repair, and the p53 signaling pathway, including CCNB1, CCNE2, E2F2, and XRCC2. Among these genes, CCNB1 [40], CCNE2 [41] and E2F2 [42] are well known for promoting cell cycle phase transitions. XRCC2 is involved in homologous recombination repair of damaged DNA [43]. The mitochondrial relocation of CCNB1/CDK1 enhances the ATP production needed for DNA damage repair, aiding cell survival and contributing to tumor resistance to DNA-damaging treatments such as chemotherapy and radiotherapy [44]. Thus, targeting HSF1 offers a multifaceted and promising anticancer therapy for DLBCL.

Given that HSF1 may form transcription complexes by binding to specific proteins, we identified PRMT5 as an HSF1 binding partner through MS and Co-IP. PRMT5 methylates arginine residues in target proteins and regulates transcription, splicing, DNA damage response, and cell metabolism [45]. Studies have shown that inhibiting PRMT5 suppresses DLBCL cell proliferation both in vitro and in patient-derived xenografts [32]. In ABC-DLBCL, BTK-NF-κB signaling induces PRMT5 transcription, whereas BCR downstream PI3K-AKT-MYC signaling upregulates PRMT5 in both ABC and germinal center B-cell-like (GCB) DLBCL. Co-inhibition of PRMT5 and AKT is lethal to DLBCL cells [32]. Lu et al. demonstrated that PRMT5 supports germinal center formation and affinity maturation by directly interacting with and methylating BCL6 at arginine 305 (R305), which is essential for the transcriptional repression of BCL6 [31]. These findings suggest that PRMT5 plays a crucial role in proliferation and progression through classic signaling pathways in B-cell lymphoma. In this study, PRMT5 knockdown inhibited proliferation and enhanced the chemosensitivity of DLBCL cells in vitro. qRT‒PCR analysis revealed that PRMT5 also regulates the expression of HSF1 target genes. Importantly, HSF1 was also found to promote lymphatic metastasis in bladder cancer via a PRMT5-WDR5-dependent transcriptional program [46]. Collectively, our results revealed that HSF1 enhances target gene expression through a PRMT5-mediated transcriptional mechanism.

While targeting gene expression using shRNA technology in vivo is currently challenging, small molecule inhibitors offer promising alternatives. Inhibitors targeting HSF1 have been developed and have shown promising results in cancer therapy, such as myeloma and leukemia [47–51]. DTHIB, a highly selective HSF1 inhibitor, exerts its function by directly binding to HSF1 and promoting its nuclear degradation [52]. The resulting loss of nuclear HSF1 reduces its occupancy at target gene promoters, thereby suppressing the transcription of HSF1-regulated oncogenes. In an acute myeloid leukemia (AML) animal model [53], DTHIB specifically suppressed the self-renewal of leukemia stem cells while sparing normal hematopoietic stem/progenitor cells. In AML, DTHIB inhibited the expression of HSP90 and reduced mitochondrial oxidative phosphorylation by downregulating SDHC, a key enzyme complex in the tricarboxylic acid cycle [53]. In this study, we found that DTHIB significantly suppressed DLBCL cell proliferation in vitro. In animal models, DTHIB administration inhibited HSF1 activity during tumor growth without causing significant toxicity. These findings support the clinical translation of HSF1-targeted therapies for the treatment of DLBCL.

In summary, our study demonstrated that overexpression of HSF1 is associated with inferior sensitivity to first-line chemotherapy and poor prognosis in DLBCL patients. HSF1 could interact with PRMT5 and regulate downstream genes, consequently promoting cell proliferation and increasing resistance to vincristine and doxorubicin. Using DTHIB to inhibit HSF1-mediated functions reverses oncogenic characteristics in DLBCL. Understanding the precise role of HSF1 in DLBCL could accelerate the development of prognostic prediction and therapeutic strategies for chemoresistant DLBCL patients.

## Supplementary information

Supplementary figures

Supplementary tables

Original western blots

## Data Availability

RNA sequencing data have been deposited to the Gene Expression Omnibus (GEO) and can be accessed using the following identifiers: GSE295838. Additional datasets used and analysed during the current study are available on reasonable request from the corresponding author, Qingqing Cai (caiqq@sysucc.org.cn).
