# Supplementary material for: Heat shock factor 1 promotes proliferation and chemoresistance in diffuse large B-cell lymphoma by enhancing the cell cycle and DNA repair
Source: Cell Death Dis. 2025 Jul 17;16(1):533. doi: 10.1038/s41419-025-07843-2 (PMC12271312; doi:10.1038/s41419-025-07843-2)
Supplement: Supplementary file 1 — Supplementary figures [file 41419_2025_7843_MOESM1_ESM.docx]

**Heat shock factor 1 promotes proliferation and chemoresistance in diffuse large B-cell lymphoma by enhancing the cell cycle and DNA repair**

**Table of contents**

[Figure S1. 1](#_Toc7488)

[Figure S2. 1](#_Toc9911)

[Figure S3. 2](#_Toc7855)

[Figure S4. 2](#_Toc26401)

[Figure S5. 3](#_Toc29805)

[Figure S6 3](#_Toc29077)

[Figure S7. 4](#_Toc16426)

[Figure S8. 5](#_Toc8882)

[Figure S9. 6](#_Toc28402)

[Figure S10 7](#_Toc21146)

[Figure S11 7](#_Toc8858)

[Figure S12. 8](#_Toc1309)

[Figure S13. 9](#_Toc23487)

[Figure S14 1](#_Toc15434)0

[Figure S15 1](#_Toc4235)1


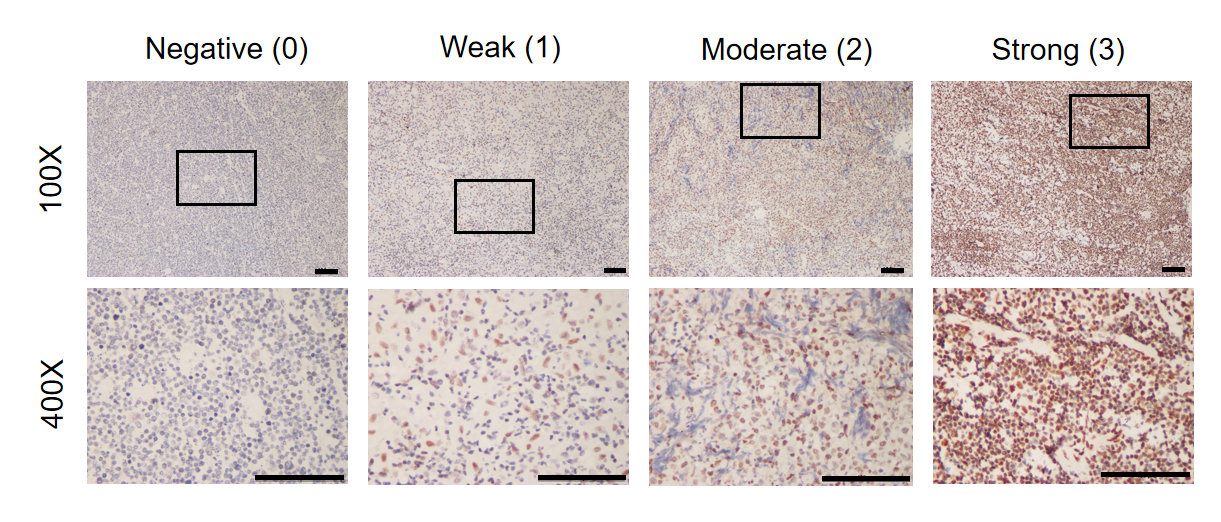


**Figure S1. Representative images of different HSF1 expression** **in DLBCL by IHC.** The level of HSF1 expression was graded according to the intensity of IHC staining: 0, no staining; 1, weak staining; 2, moderate staining; and 3, strong staining. Scale bars: 100 μm.


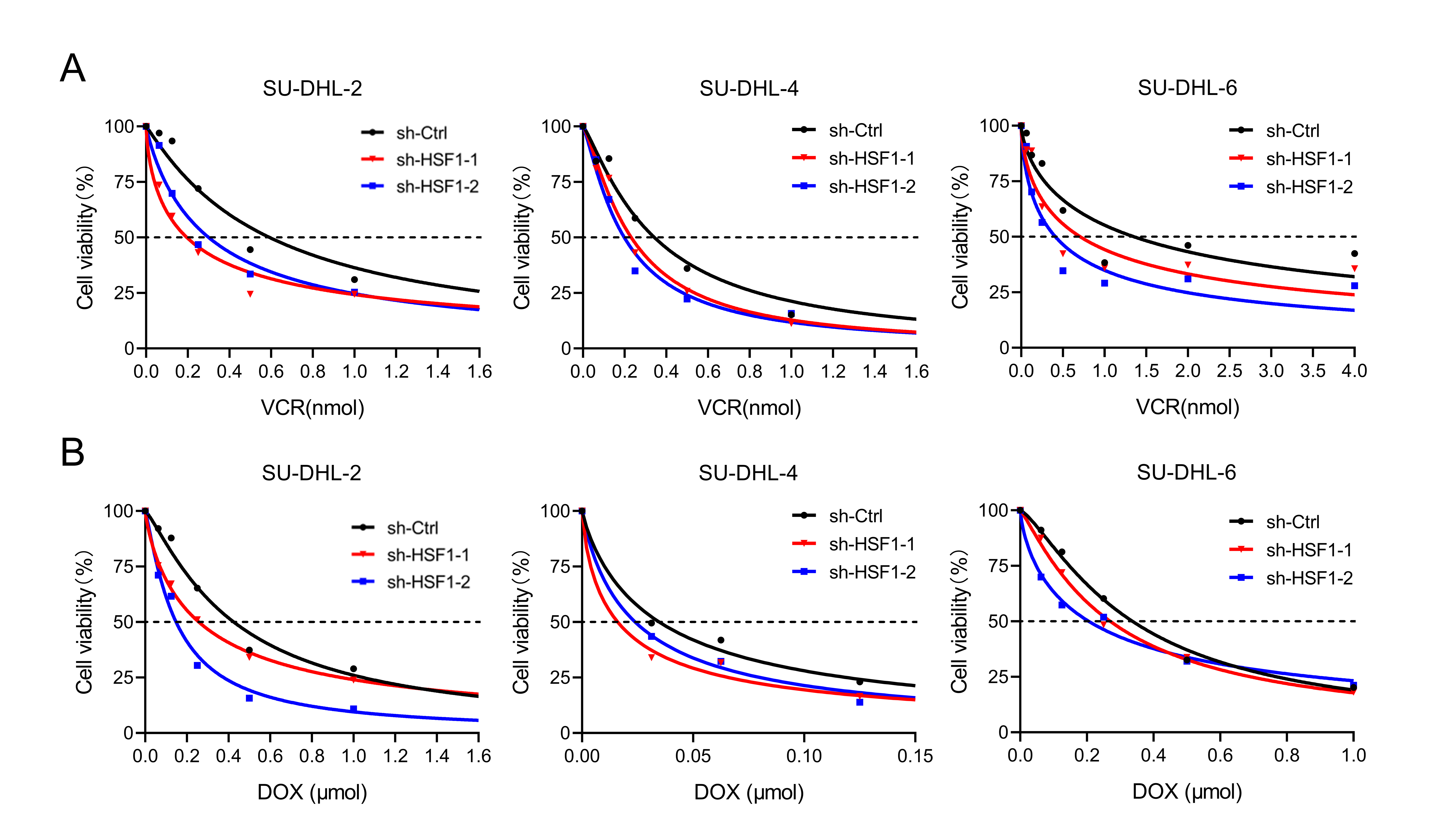


## Figure S2. HSF1 knockdown increases the chemosensitivity of DLBCL cells to vincristine and doxorubicin. The viability of SU-DHL-2, SU-DHL-4 and SU-DHL-6 cells with HSF1 knockdown via shRNA combined with vincristine (**A**) or doxorubicin (**B**), as determined by a CCK8 assay.


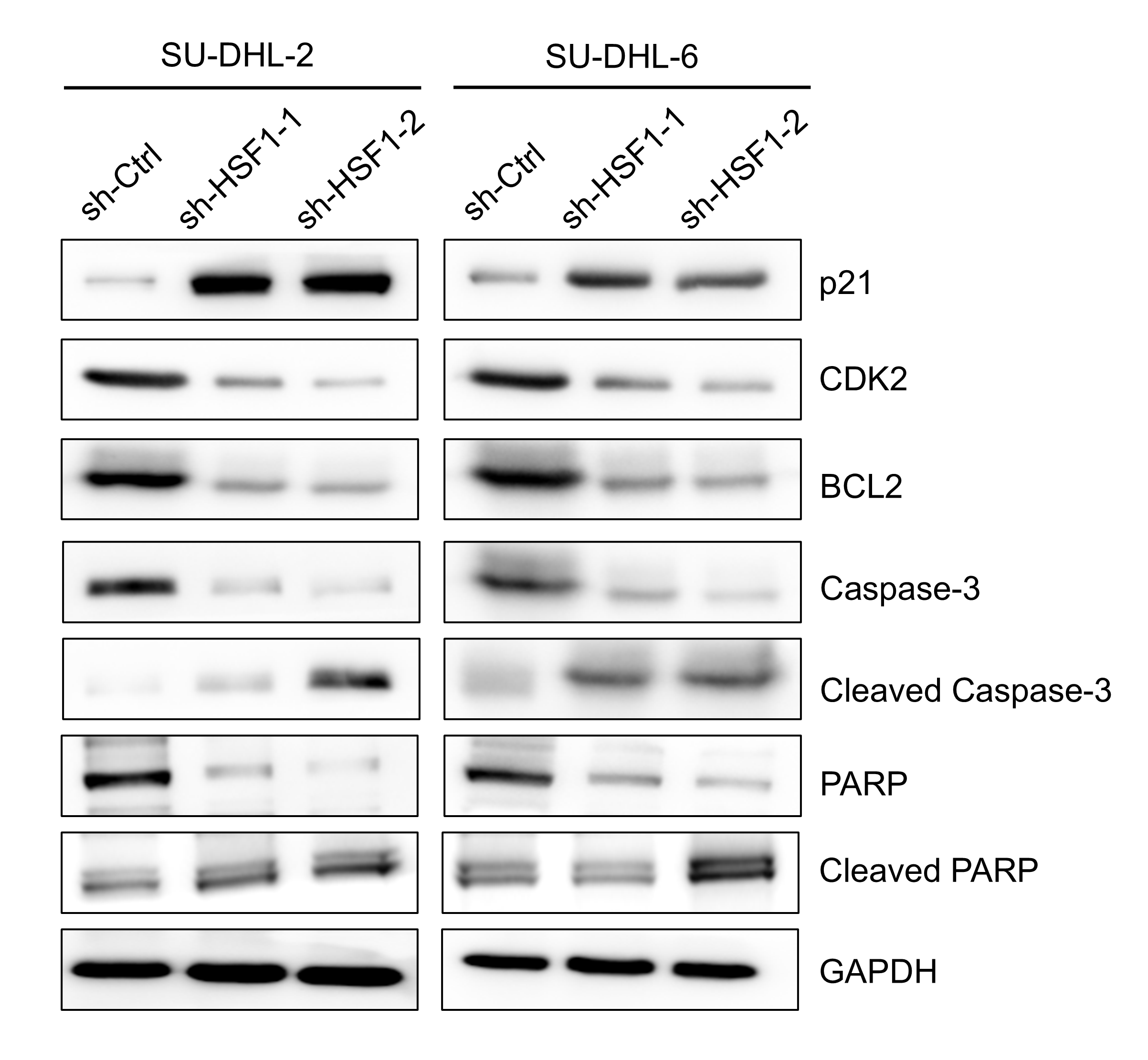


**Figure S3.** Western blot analysis of p21, CDK2, BCL2, Caspase-3, cleaved Caspase-3, PARP and cleaved PARP proteins after HSF1 knockdown in SU-DHL-2 and SU-DHL-6 cells. GAPDH was used as a loading control.


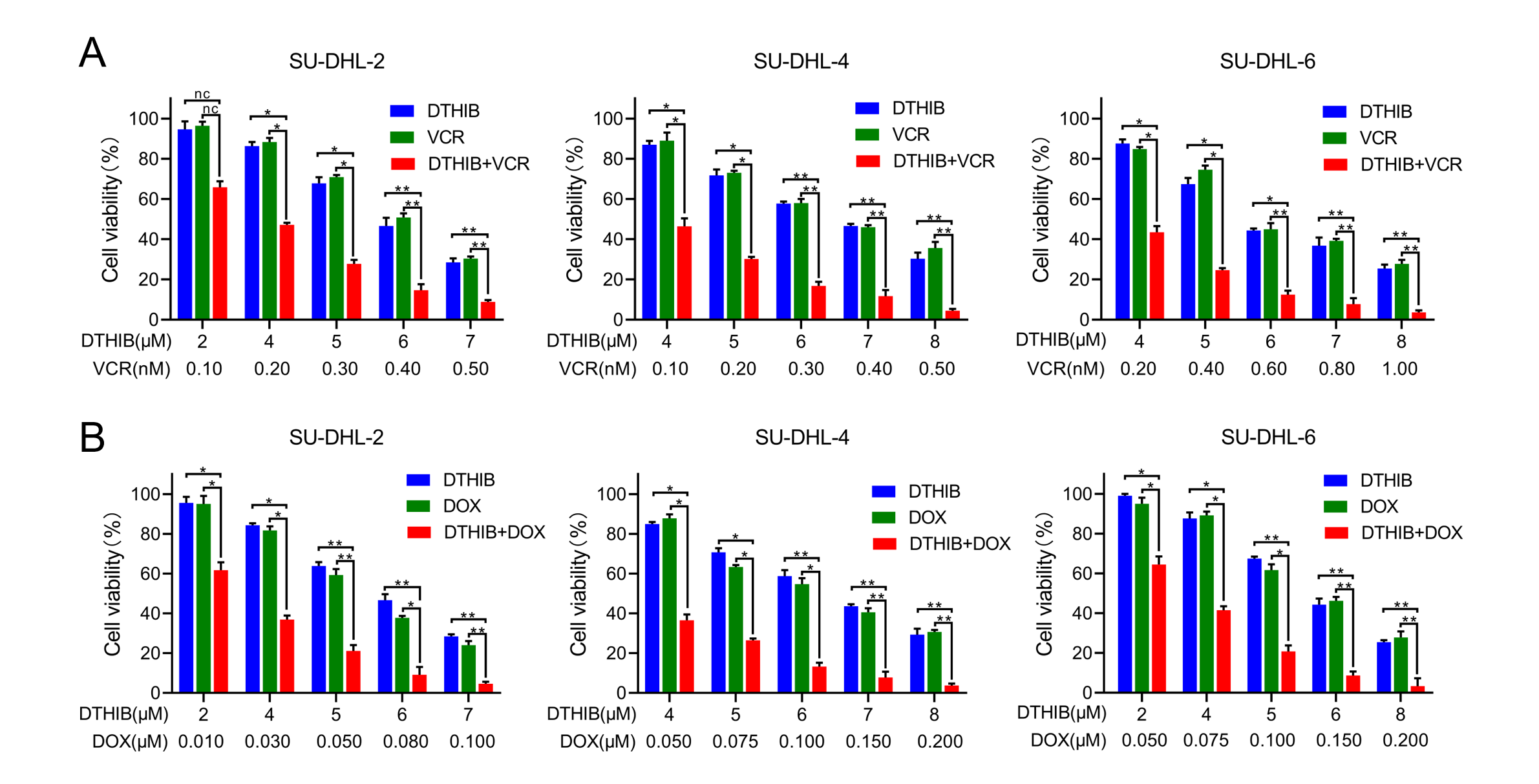


**Figure S4.** Cell viabilities of SU-DHL-2, SU-DHL-4 and SU-DHL-6 cells treated with combination of DTHIB and vincristine (**A**) and combination of DTHIB and doxorubicin (**B**). **p* < 0.05, ***p* < 0.01.


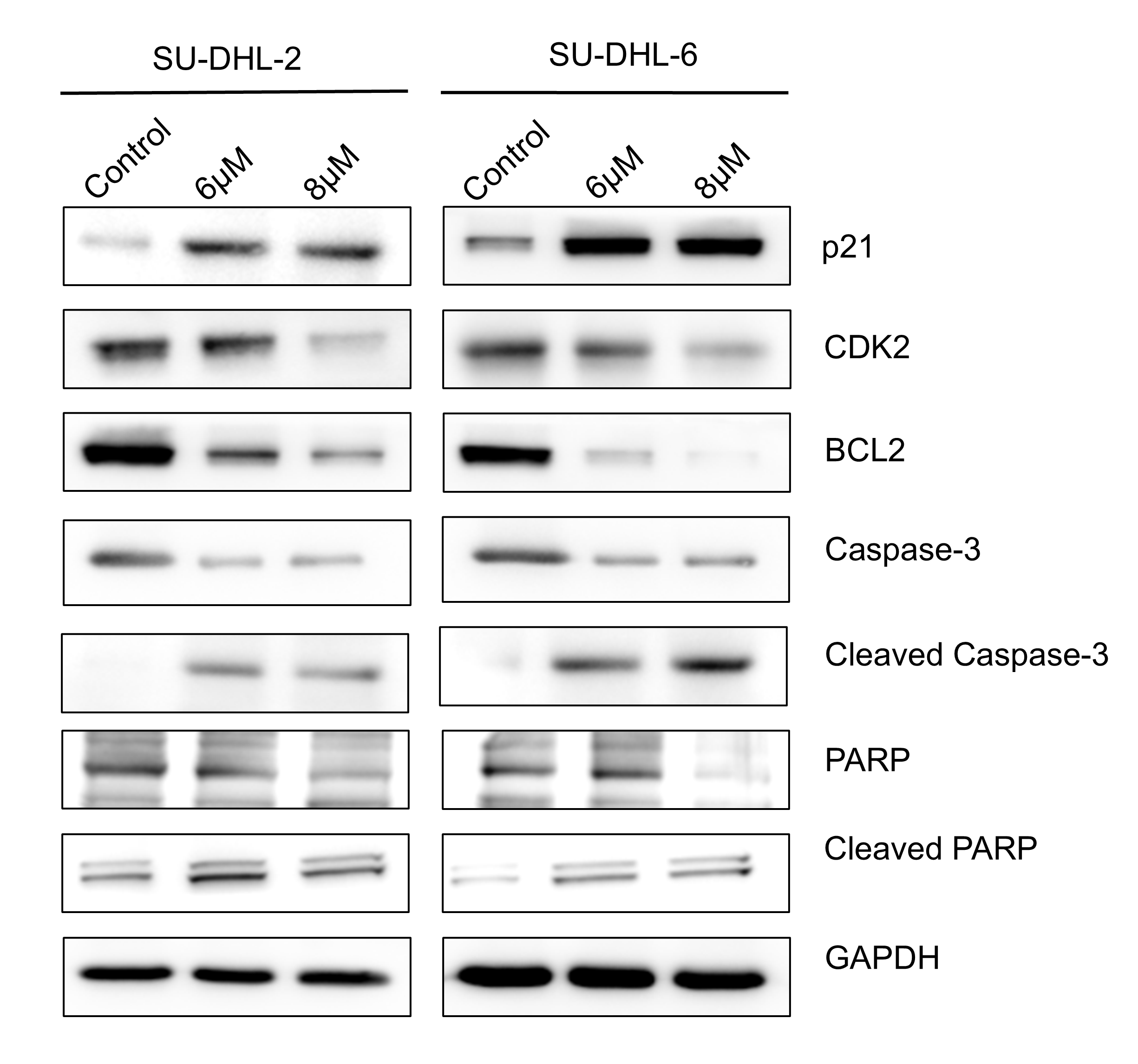


**Figure S5.** Western blot analysis of p21, CDK2, BCL2, Caspase-3, cleaved Caspase-3, PARP and cleaved PARP proteins in SU-DHL-2 and SU-DHL-6 cells after DTHIB treatment. GAPDH was used as a loading control.


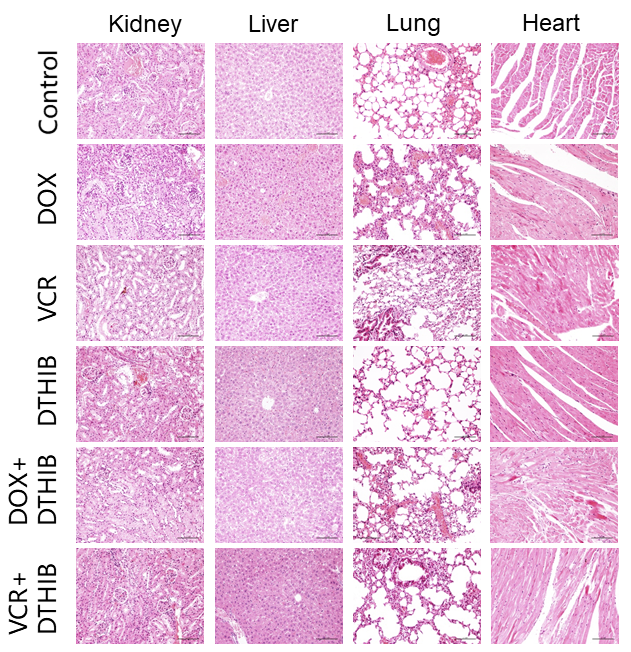


**Figure S6.** H&E staining of kidney, liver, lung, and heart from the mice in indicated groups. Scale bars: 100 μm.


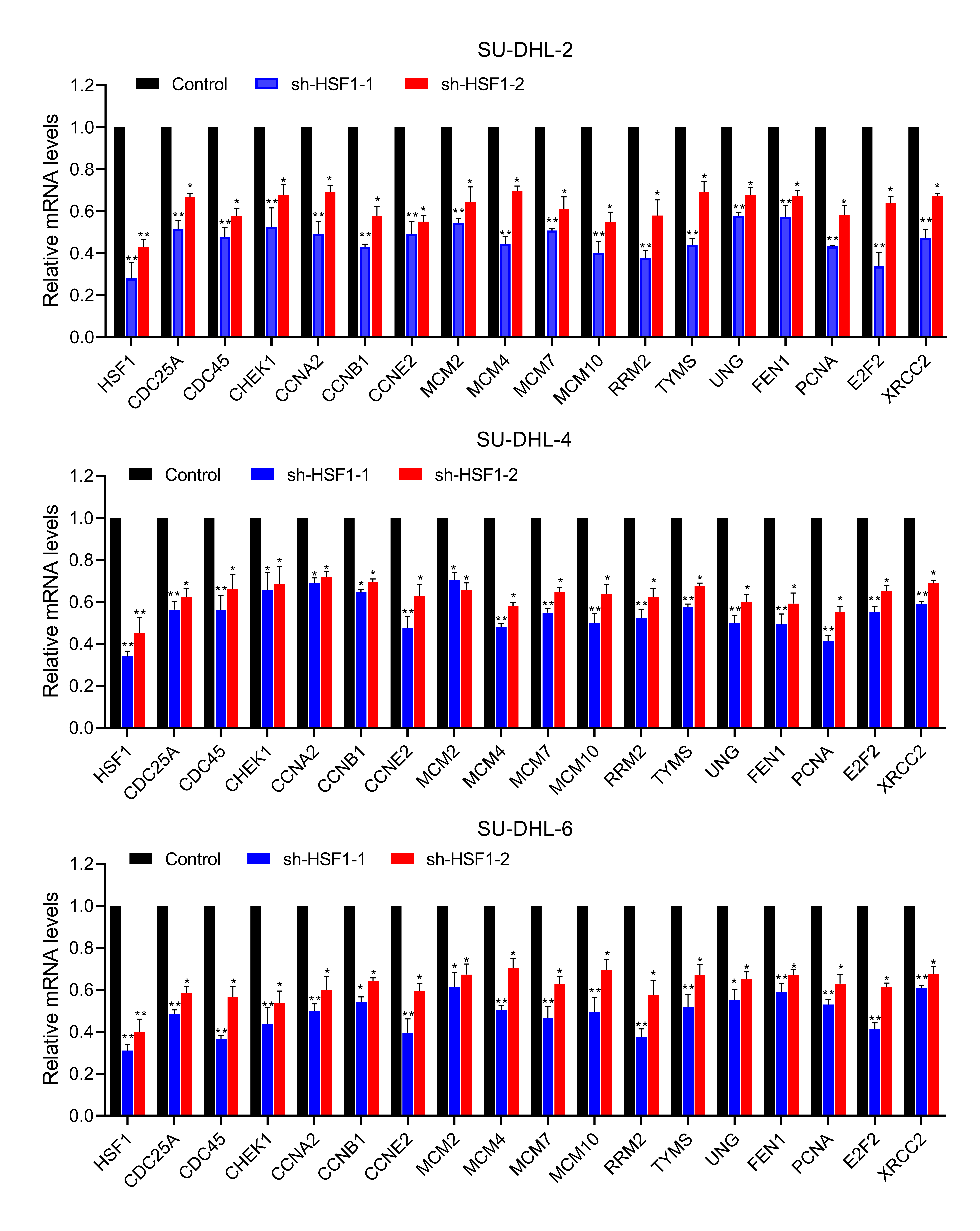


**Figure S7.** Validation of candidate downregulated genes by qRT‒PCR in SU-DHL-2, SU-DHL-4 and SU-DHL-6. The error bars represent standard deviations of three independent experiments. **p* < 0.05, ***p* < 0.01.


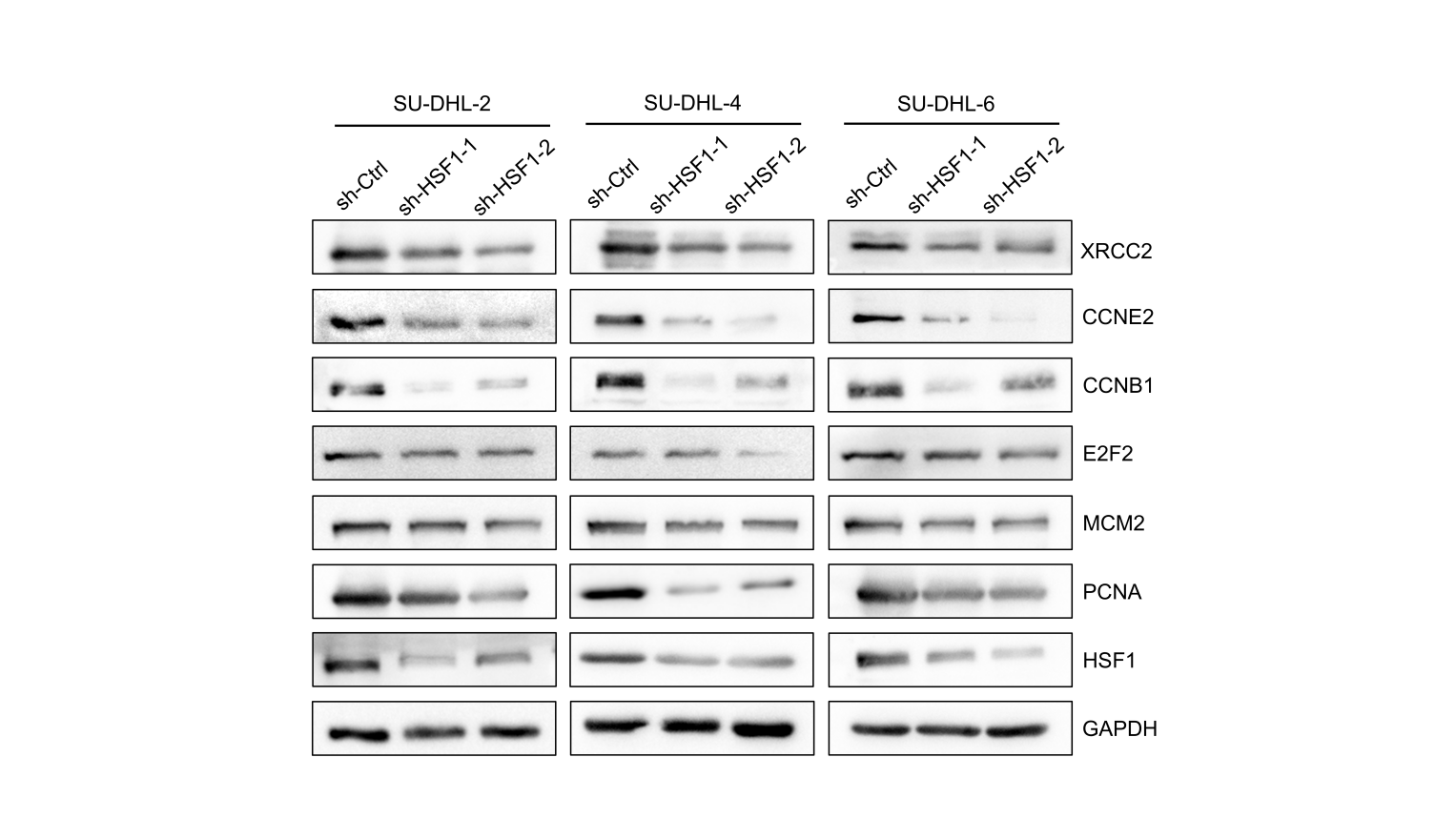


## Figure S8. Validation of candidate downregulated genes by Western blotting in SU-DHL-2, SU-DHL-4 and SU-DHL-6. GAPDH were used as internal controls.


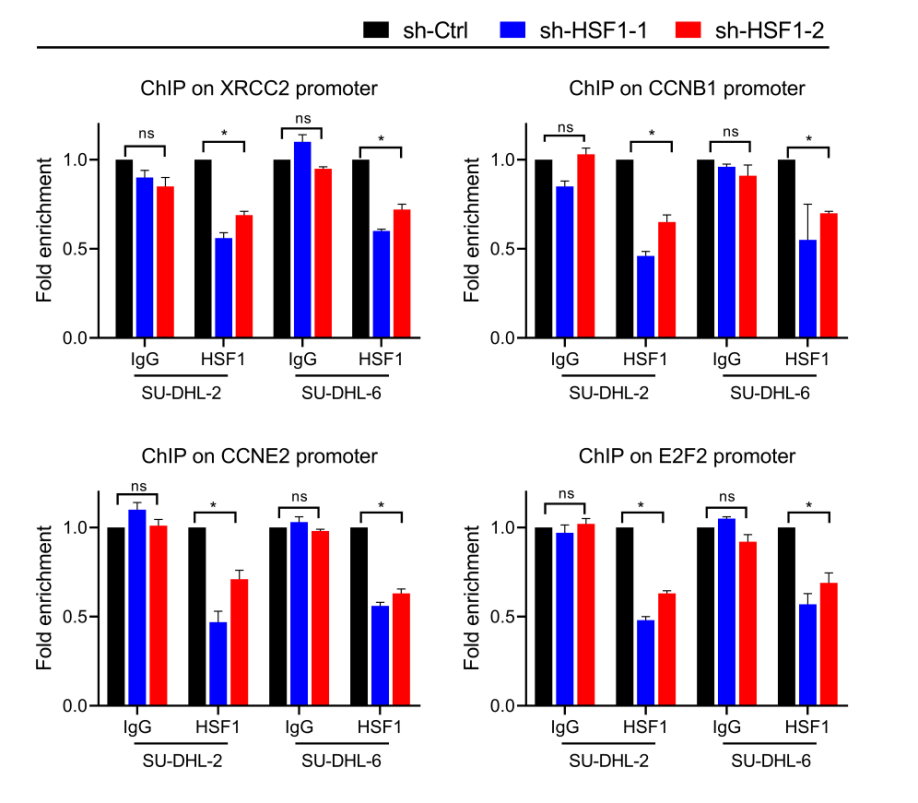


## Figure S9. ChIP analysis of negative control IgG and HSF1 enrichment at the promoters of HSF1 target genes in SU-DHL-2 and SU-DHL-6 cells transfected with HSF1 or Ctrl shRNA. The values are normalized to the input values and are presented as the means ± SDs. The error bars represent the standard deviations of three independent experiments. **p* < 0.05; ns, not statistically significant.


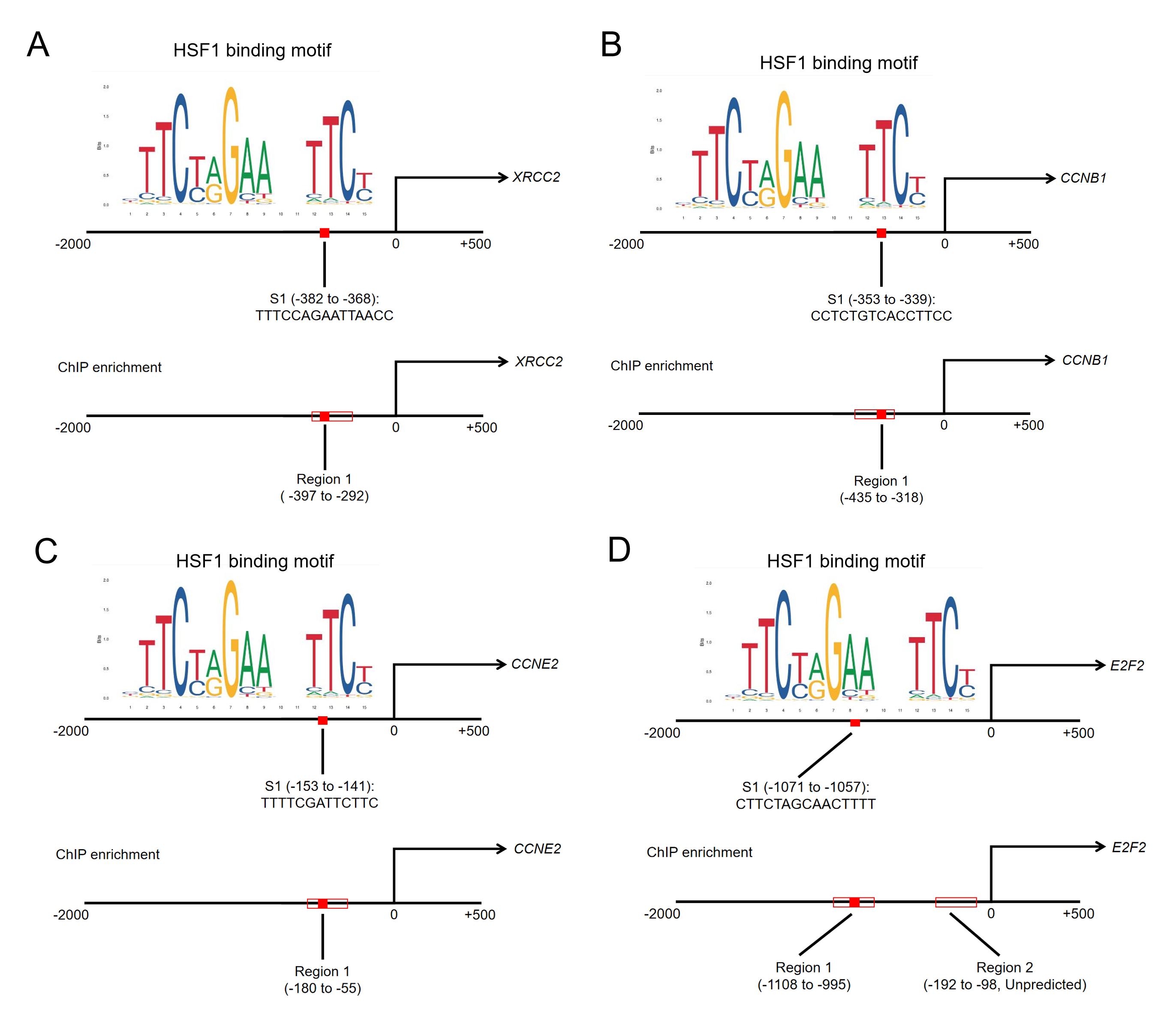


**Figure S10.** Schematic illustration of potential HSF1 targeting sites predicted by JASPAR and chromatin immunoprecipitation (ChIP)-qPCR amplification regions in the XRCC2 (**A**), CCNB1 (**B**), CCNE2 (**C**), and E2F2 (**D**) promoters.


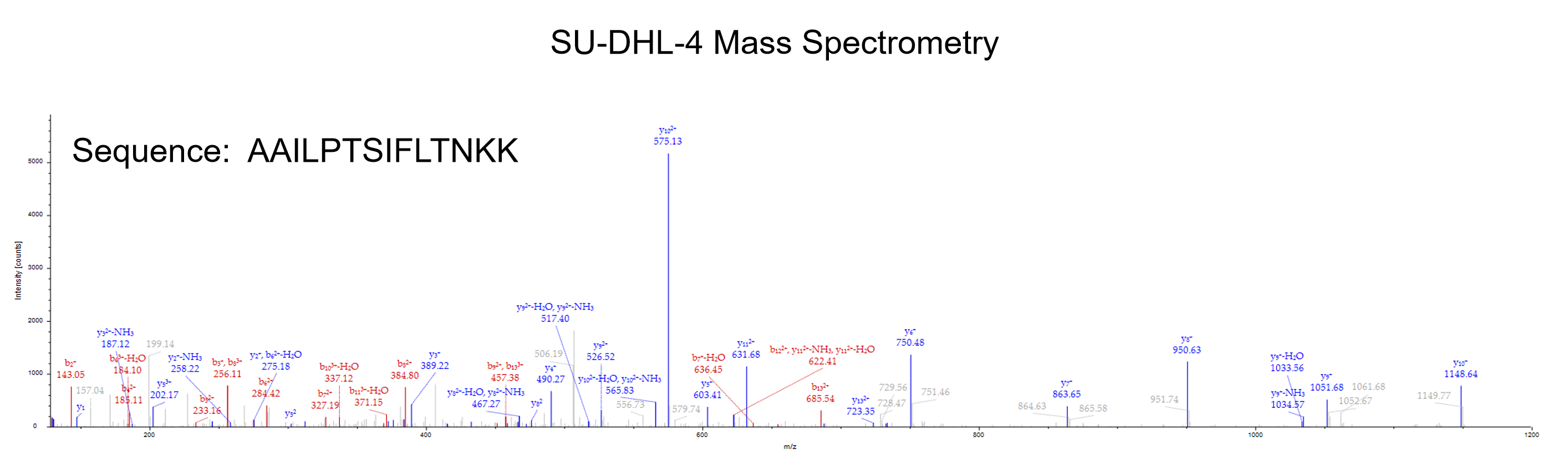


## Figure S11. Mass spectrometry (MS) identification of HSF1-interacting proteins in SU-DHL-4 cells.

**
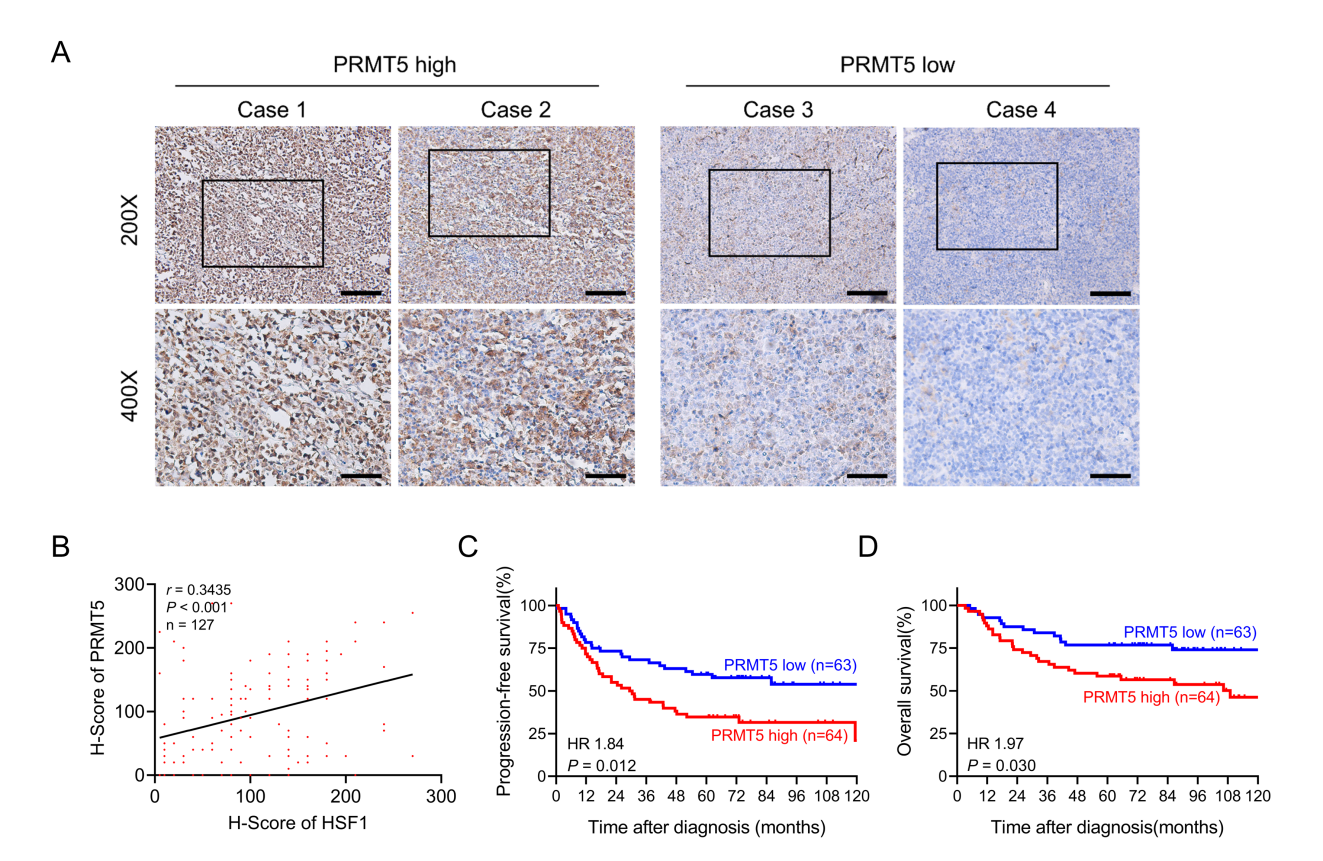
**

**Figure S12. PRMT5 was positively associated with HSF1 expression and poor prognosis in DLBCL patients. A.** Representative images of different PRMT5 expression patterns in DLBCL samples from the SYSUCC patient cohort determined via IHC. **B.** Pearson correlations between the expression of HSF1 and PRMT5 in the SYSUCC patient cohort. **C‒D.** Kaplan‒Meier curves for progression-free survival (C) and overall survival (D) of DLBCL patients with high (H-score ≥ 90) *vs.* low expression of PRMT5 (H-score < 90).


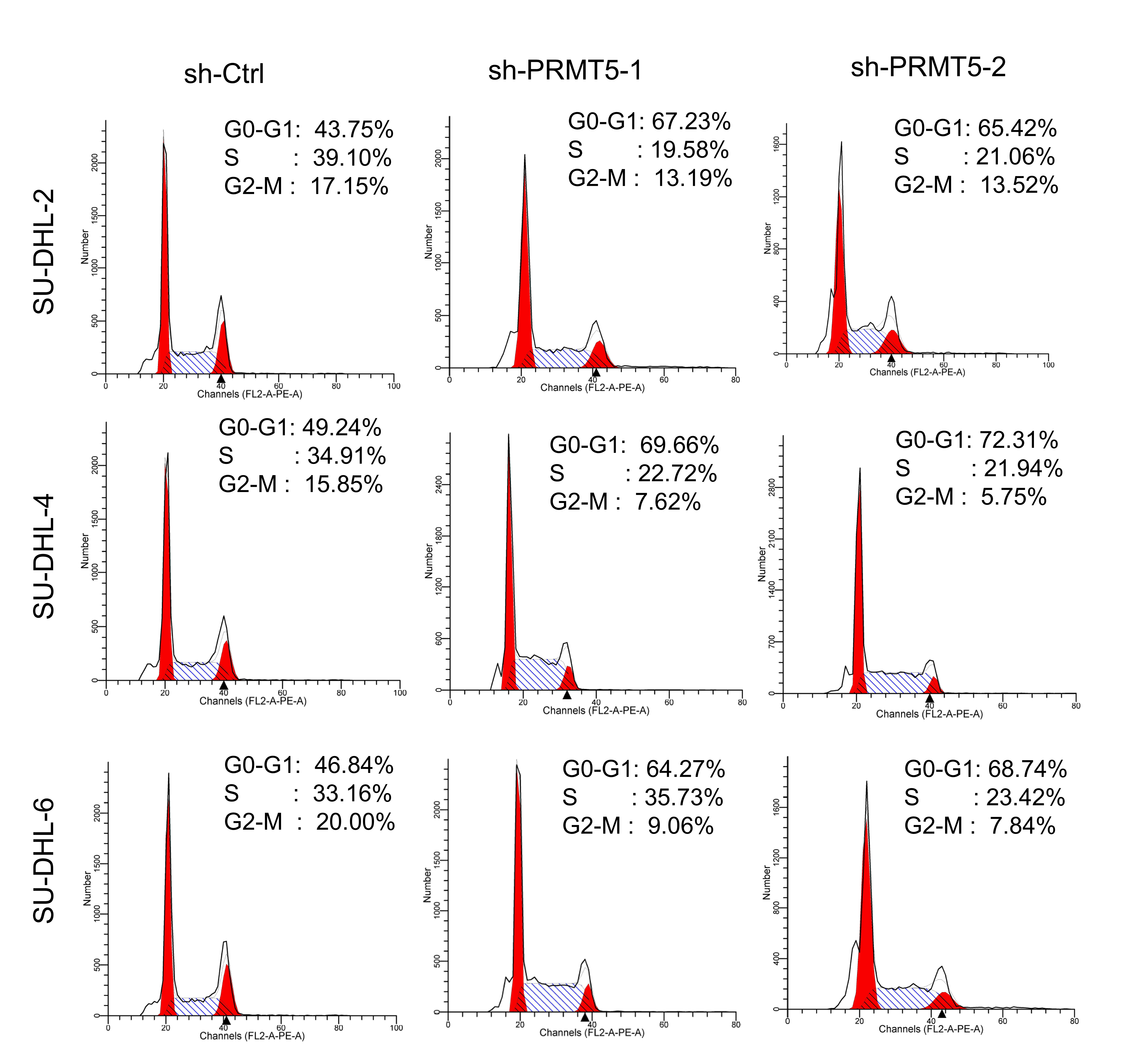


Figure S13. Representative images of the cell cycle in SU-DHL-2, SU-DHL-4 and SU-DHL-6 cells transfected with PRMT5 or Ctrl shRNA, analysed by flow cytometry analysis.

**
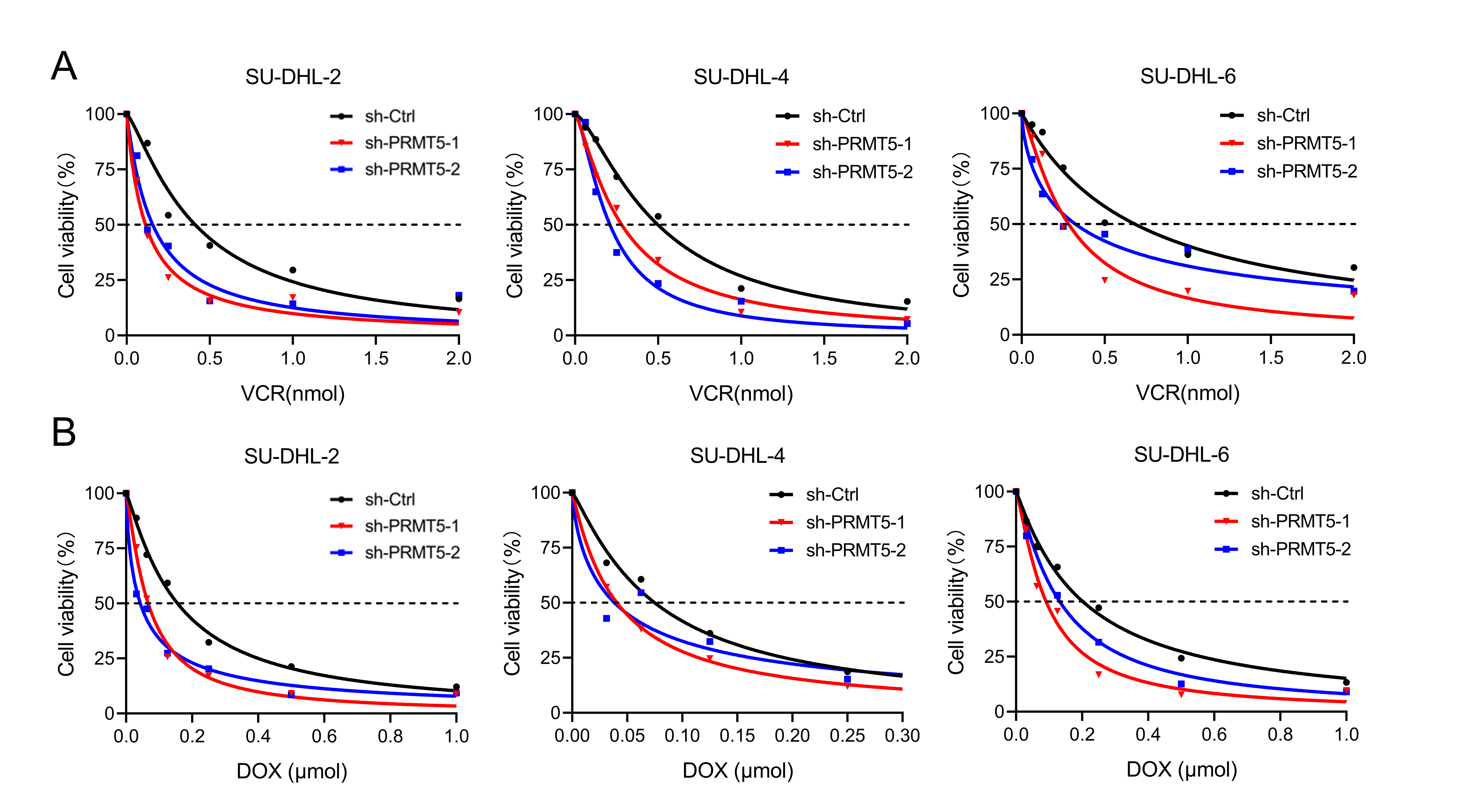
**

Figure S14. PRMT5 knockdown increases the chemosensitivity of DLBCL cells to vincristine and doxorubicin. The viability of SU-DHL-2, SU-DHL-4 and SU-DHL-6 cells with PRMT5 knockdown via shRNA combined with vincristine (**A**) or doxorubicin (**B**), as determined by a CCK8 assay.


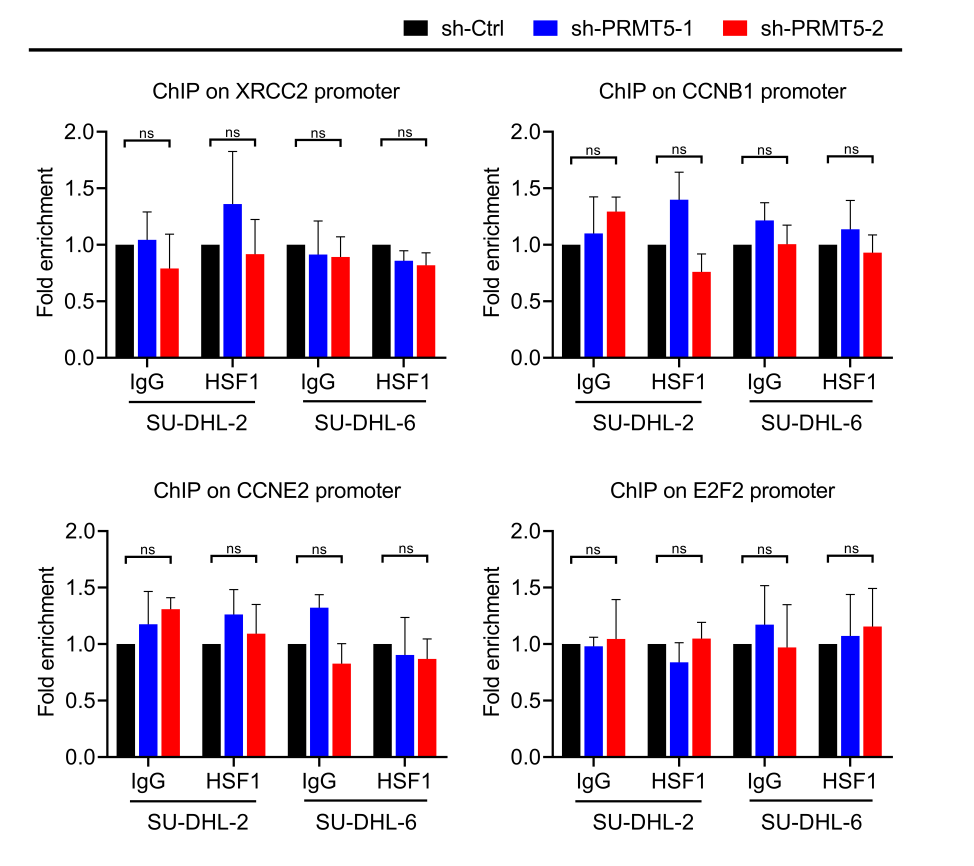


## Figure S15. ChIP analysis of negative control IgG and HSF1 enrichment at the promoters of HSF1 target genes in SU-DHL-2 and SU-DHL-6 cells transfected with PRMT5 or Ctrl shRNA. The error bars represent the standard deviations of three independent experiments. **p* < 0.05; ns, not statistically significant.
