# Supplementary material for: Heat shock factor 1 promotes proliferation and chemoresistance in diffuse large B-cell lymphoma by enhancing the cell cycle and DNA repair
Source: Cell Death Dis. 2025 Jul 17;16(1):533. doi: 10.1038/s41419-025-07843-2 (PMC12271312; doi:10.1038/s41419-025-07843-2)
Supplement: Supplementary file 2 — Supplementary tables [file 41419_2025_7843_MOESM2_ESM.docx]

**Heat shock factor 1 promotes proliferation and chemoresistance in diffuse large B-cell lymphoma by enhancing the cell cycle and DNA repair**

**Table of Contents**

[Table S1. 1](#_Toc13537)

[Table S2. 2](#_Toc16072)

[Table S3. 3](#_Toc18497)

[Table S4. 4](#_Toc26935)

[Table S5. 4](#_Toc30054)

[Table S6. 6](#_Toc19992)

[Table S7. 6](#_Toc11506)

[Table S8. 7](#_Toc29576)

##

## Table S1. The clinicopathological characteristics of patient cohort from Sun Yat-sen University Cancer Center.

| Variables |  | Number of cases (%) |
| --- | --- | --- |
| Age | Median (years) | 53.5 |
|  | < 60 years | 129 (65.8%) |
|  | ≥ 60 years | 67 (34.2%) |
| Sex | Male | 116 (59.2%) |
|  | Female | 80 (40.8%) |
| ECOG PS | 0-1 | 161 (82.1%) |
|  | ≥ 2 | 35 (17.9%) |
| Elevated LDH | Yes | 121 (61.7%) |
|  | No | 75 (38.3%) |
| Ann Arbor stage | I - II | 69 (35.1%) |
|  | III - IV | 127 (64.9%) |
| Number of extranodal site | 0-1 | 144 (73.4%) |
|  | > 1 | 52 (26.6%) |
| IPI score | 0-2 | 131 (66.8%) |
|  | 3-5 | 65 (33.2%) |
| COO class | Non-GCB | 122 (62.2%) |
|  | GCB | 74 (37.8%) |
| HSF1 | High | 98 (50%) |
|  | Low | 98 (50%) |

ECOG PS, Eastern Cooperative Oncology Group performance status; LDH, lactate dehydrogenase; IPI, International Prognostic Index; COO: cell of origin; GCB, general center B-cell-like; HSF1, heat shock factor 1.

## Table S2. The clinicopathological characteristics of patients from two GEO datasets.

| Variables |  | GSE10846  (n=233) |  | GSE117556  (n=469) |
| --- | --- | --- | --- | --- |
|  |  | Number of cases (%) |  | Number of cases (%) |
| Age (years) | <60  ≥60 | 111 (47.6%)  122 (52.4%) | <60  ≥60 | 150 (32.0%)  319 (68.0%) |
| Sex | Male  Female | 134 (57.5%)  99 (42.5%) | Male  Female | 264 (56.3%)  205 (43.7%) |
| ECOG PS | 0-1  ≥ 2 | 158 (75.2%)  52 (24.8%) | 0-1  ≥ 2 | 417 (88.9%)  52 (11.1%) |
| LDH | ≤ ULN  > ULN | 99 (51.6%)  93 (48.4%) | <500U/L  ≥500U/L | 298 (63.5%)  171 (36.5%) |
| Ann Arbor stage | I-II  III-IV | 105 (46.5%)  121 (53.5%) | I-II  III-IV | 146 (31.3%)  321 (48.7%) |
| Extranodal site | 0-1  ≥ 2 | 174 (85.3%)  30 (14.7%) | Yes  No | 198 (42.2%)  271 (57.8%) |
| IPI score | 0-1  2-3  4-5 | 75 (41.2%)  83 (45.6%)  24 (13.2%) | 0-2  3-5 | 239 (51.0%)  230 (49.0%) |
| COO class | GCB  ABC | 107 (53.5%)  93 (46.5%) | GCB  ABC | 277 (68.2%)  129 (31.8%) |
| HSF1 | High  Low | 156 (67.0%)  77 (33.0%) | High  Low | 319 (68.0%)  150 (32.0%) |

ECOG PS, Eastern Cooperative Oncology Group performance status; LDH, lactate dehydrogenase; ULN, upper limit of normal; IPI, International Prognostic Index; COO: cell of origin; GCB, general center B-cell-like; ABC, activated B cell; HSF1, heat shock factor 1.

## Table S3. Antibodies used in this study.

| **Applification** | **Proteins** | **Vendor** | **Cat**  **number** | **Dilution for**  **usage** |
| --- | --- | --- | --- | --- |
| Western blotting | HSF1 | Abcam | Ab52757 | 1:10000 |
| Western blotting | PRMT5 | Proteintech | 18436-1-AP | 1:1000 |
| Western blotting | Cyclin B1 | CST | #4138 | 1:1000 |
| Western blotting | Cyclin E2 | CST | #4132 | 1:1000 |
| Western blotting | XRCC2 | Proteintech | 20285-1-AP | 1:1000 |
| Western blotting | E2F2 | Abcam | Ab235837 | 1:1000 |
| Western blotting | MCM2 | CST | #4007 | 1:1000 |
| Western blotting | PCNA | Proteintech | 10205-2-AP | 1:10000 |
| Western blotting | p21 | Immunoway | YM8364 | 1:2000 |
| Western blotting | CDK2 | Immunoway | YM8146 | 1:2000 |
| Western blotting | Bcl-2 | Immunoway | YM8319 | 1:2000 |
| Western blotting | Caspase-3 | CST | 14220 | 1:1000 |
| Western blotting | Cleaved Caspase-3 | CST | 9664 | 1:1000 |
| Western blotting | PARP | CST | 9542 | 1:1000 |
| Western blotting | Cleaved PARP | CST | 5625 | 1:1000 |
| Western blotting | GAPDH | Proteintech | 60004-1-Ig | 1:20000 |
| Immunohistochemistry | HSF1 | Abcam | Ab52757 | 1:400 |
| Immunohistochemistry | PRMT5 | CST | 79998S | 1:1000 |
| Immunoprecipitation | HSF1 | CST | #12972 | 1:50 |
| Immunoprecipitation | PRMT5 | Proteintech | 18436-1-AP | 1:50 |
| Immunoprecipitation | IgG | CST | #3900 | 1:500 |
| Chromatin immunoprecipitation | HSF1 | CST | #12972 | 1:50 |
| Chromatin immunoprecipitation | RNA pol II | Abcam | Ab817 | 1:200 |
| Chromatin immunoprecipitation | IgG | CST | #3900 | 1:500 |

##

## Table S4. The shRNA sequences used in this study.

| Primer Name | Sequence 5’-3’ |
| --- | --- |
| sh-Ctrl | CAACAAGATGAAGAGCACCAA |
| sh-HSF1-1 | TAGCCTGCCTGGACAAGAA |
| sh-HSF1-2 | GTGCTGCCCAAGTACTTCA |
| sh-PRMT5-1 | GCTAATTGTGGGAAAGCTT |
| sh-PRMT5-2 | CCAGCAGGCCATCTATAAA |

## Table S5. The primers used in real time qPCR in this study.

| Primer Name | Sequence 5’-3’ |
| --- | --- |
| HSF1 Forward | CAGCTCTGGACCCATCATCTC |
| HSF1 Reverse | GGATAGGGGCCTCTCGTCTAT |
| PRMT5 Forward | CTAGACCGAGTACCAGAAGAGG |
| PRMT5 Reverse | CAGCATACAGCTTTATCCGCCG |
| CDC25A Forward | TCTGGACAGCTCCTCTCGTCAT |
| CDC25A Reverse | ACTTCCAGGTGGAGACTCCTCT |
| CDC45 Forward | TGGATGCTGTCCAAGGACCTGA |
| CDC45 Reverse | CAGGACACCAACATCAGTCACG |
| CHEK1 Forward | GTGTCAGAGTCTCCCAGTGGAT |
| CHEK1 Reverse | GTTCTGGCTGAGAACTGGAGTAC |
| CCNA2 Forward | CTCTACACAGTCACGGGACAAAG |
| CCNA2 Reverse | CTGTGGTGCTTTGAGGTAGGTC |
| CCNB1 Forward | TAAGGCGAAGATCAACATGG |
| CCNB1 Reverse | TTACCAATGTCCCCAAGAGC |
| CCNE2 Forward | CTTACGTCACTGATGGTGCTTGC |
| CCNE2 Reverse | CTTGGAGAAAGAGATTTAGCCAGG |
| MCM2 Forward | TGCCAGCATTGCTCCTTCCATC |
| MCM2 Reverse | AAACTGCGACTTCGCTGTGCCA |
| MCM4 Forward | CTTGCTTCAGCCTTGGCTCCAA |
| MCM4 Reverse | GTCGCCACACAGCAAGATGTTG |
| MCM7 Forward | GCCAAGTCTCAGCTCCTGTCAT |
| MCM7 Reverse | CCTCTAAGGTCAGTTCTCCACTC |
| MCM10 Forward | TCAAGGAACTGATGGACCTGCC |
| MCM10 Reverse | CTCCAACATCCGCTGCTTCTGT |
| RRM2 Forward | CTGGCTCAAGAAACGAGGACTG |
| RRM2 Reverse | CTCTCCTCCGATGGTTTGTGTAC |
| TYMS Forward | GGTGTTTTGGAGGAGTTGCTGTG |
| TYMS Reverse | GGAGAATCCCAGGCTGTCCAAA |
| UNG Forward | CCACACCAAGTCTTCACCTGGA |
| UNG Reverse | CCGTGAGCTTGATTAGGTCCATG |
| FEN1 Forward | ACTAAGCGGCTGGTGAAGGTCA |
| FEN1 Reverse | GCAGCATAGACTTTGCCAGCCT |
| PCNA Forward | CAAGTAATGTCGATAAAGAGGAGG |
| PCNA Reverse | GTGTCACCGTTGAAGAGAGTGG |
| E2F2 Forward | CTCTCTGAGCTTCAAGCACCTG |
| E2F2 Reverse | CTTGACGGCAATCACTGTCTGC |
| XRCC2 Forward | TCTGTTTGCTGATGAAGATTCACC |
| XRCC2 Reverse | CATCGTGCTGTTAGGTGATAAAGC |
| GPADH Forward | CAAGGCTGAGAACGGGAAG |
| GPADH Reverse | TGAAGACGCCAGTGGACTC |

## Table S6. The primers used in ChIP-real time qPCR in this study.

| Primer Name | Sequence 5’-3’ |
| --- | --- |
| E2F2-P Forward | CACCAGGCAAGAAATCAGACC |
| E2F2-P Reverse | ACATTCCTCATCACTCCGGT |
| CCNB1-P Forward | CAGACCACGTGAGAGCCTG |
| CCNB1-P Reverse | ATTTAAACCCCGCACTGCTC |
| XRCC2-P Forward | GCACACCCTATTGCGCATG |
| XRCC2-P Reverse | ACTCTACGGCCAGTCAAACC |
| CCNE2-P Forward | CTTGGGCAAAGCTCCTACAG |
| CCNE2-P Reverse | TTGAGGCCCGGGAAATAGC |
| Negative control F | GTAATCAGGAAACTGCATAC |
| Negative control R | CTCAAGACTCAATAGTGATC |

## Table S7. Univariate and multivariate analyses of PFS in patient cohort from Sun Yat-sen University Cancer Center.

| Variables | PFS | | | | |
| --- | --- | --- | --- | --- | --- |
|  | Univariate | |  | Multivariate | |
|  | HR (95%CI) | *P* |  | HR (95%CI) | *P* |
| Age (years)  ≥60 / <60 | 1.31  (0.87-1.96) | 0.197 |  |  |  |
| Gender  Male / Female | 0.91  (0.61-1.37) | 0.649 |  |  |  |
| ECOG PS  ≥2 / 0-1 | 1.55  (0.75-3.17) | 0.153 |  |  |  |
| Ann Arbor stage  III-IV / I-II | 2.22  (1.37-3.60) | 0.001 |  | 1.55  (0.84-2.85) | 0.158 |
| COO class  non-GCB / GCB | 1.65  (1.04-2.60) | 0.032 |  | 1.42  (0.87-2.33) | 0.162 |
| Serum LDH  Elevated / Normal | 2.05  (1.36-3.10) | 0.001 |  | 1.38  (0.77-2.49) | 0.278 |
| Extranodal involvement  ≥2 / 0-1 | 1.57  (1.01-2.45) | 0.046 |  | 1.78  (0.95-3.36) | 0.073 |
| IPI score  3-5 / 0-2 | 2.28  (1.47-3.54) | <0.001 |  | 2.20  (1.03-4.71) | 0.043 |
| HSF1  High / Low | 1.71  (1.14-2.56) | 0.008 |  | 1.51  (0.95-2.41) | 0.082 |

HR, Hazard ratios; ECOG PS, Eastern Cooperative Oncology Group performance status; LDH, lactate dehydrogenase; COO: cell of origin; GCB, germinal center B-cell-like; IPI, International Prognostic Index; HSF1, heat shock factor 1.

## Table S8. Univariate and multivariate analyses of OS in patient cohort from Sun Yat-sen University Cancer Center.

| Variables | OS | | | | |
| --- | --- | --- | --- | --- | --- |
|  | Univariate | |  | Multivariate | |
|  | HR (95%CI) | *P* |  | HR (95%CI) | *P* |
| Age (years)  ≥60 / <60 | 1.66  (0.99-2.78) | 0.054 |  |  |  |
| Gender  Male / Female | 0.94  (0.56-1.57) | 0.803 |  |  |  |
| ECOG PS  ≥2 / 0-1 | 1.76  (0.73-4.25) | 0.112 |  |  |  |
| Ann Arbor stage  III-IV / I-II | 2.95  (1.52-5.76) | 0.001 |  | 2.00  (0.91-4.42) | 0.085 |
| COO class  non-GCB / GCB | 1.62  (0.89-2.96) | 0.117 |  |  |  |
| Serum LDH  Elevated / Normal | 1.85  (1.10-3.12) | 0.021 |  | 1.28  (0.64-2.55) | 0.490 |
| Extranodal involvement  ≥2 / 0-1 | 1.38  (0.77-2.46) | 0.282 |  |  |  |
| IPI score  3-5 / 0-2 | 2.54  (1.45-4.46) | <0.001 |  | 1.94  (0.90-4.19) | 0.092 |
| HSF1  High / Low | 1.69  (1.01-2.83) | 0.045 |  | 1.26  (0.70-2.27) | 0.436 |

HR, Hazard ratios; ECOG PS, Eastern Cooperative Oncology Group performance status; LDH, lactate dehydrogenase; COO: cell of origin; GCB, germinal center B-cell-like; IPI, International Prognostic Index; HSF1, heat shock factor 1.
