# Supplementary figures and images for: Heat shock factor 1 promotes proliferation and chemoresistance in diffuse large B-cell lymphoma by enhancing the cell cycle and DNA repair
Source: Cell Death Dis. 2025 Jul 17;16(1):533. doi: 10.1038/s41419-025-07843-2 (PMC12271312; doi:10.1038/s41419-025-07843-2)

Figure 2A

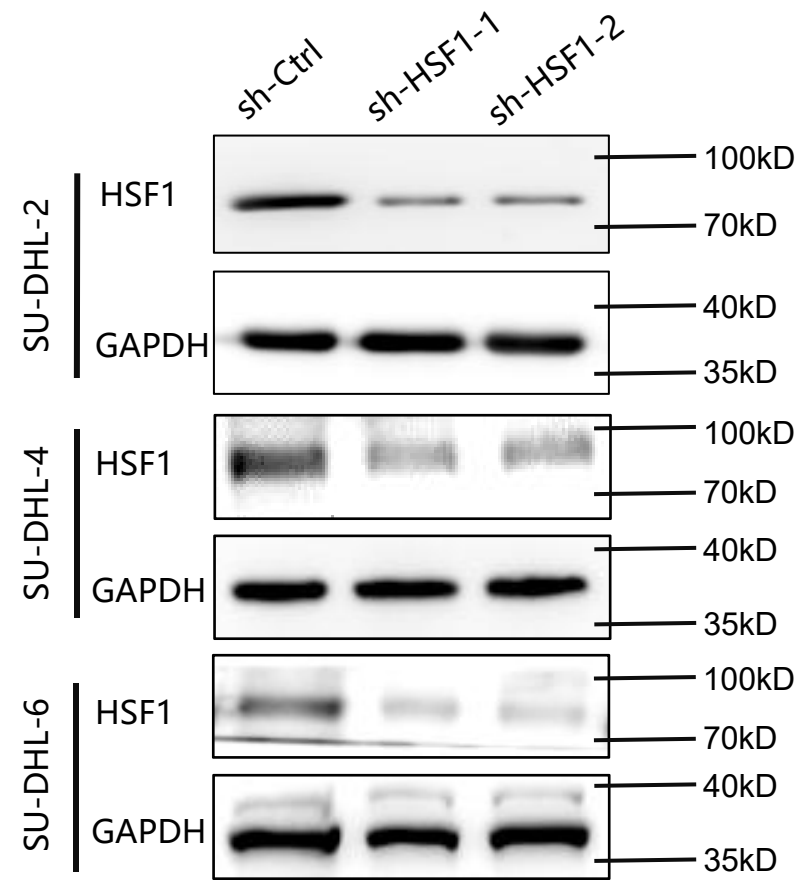

### Figure 6F

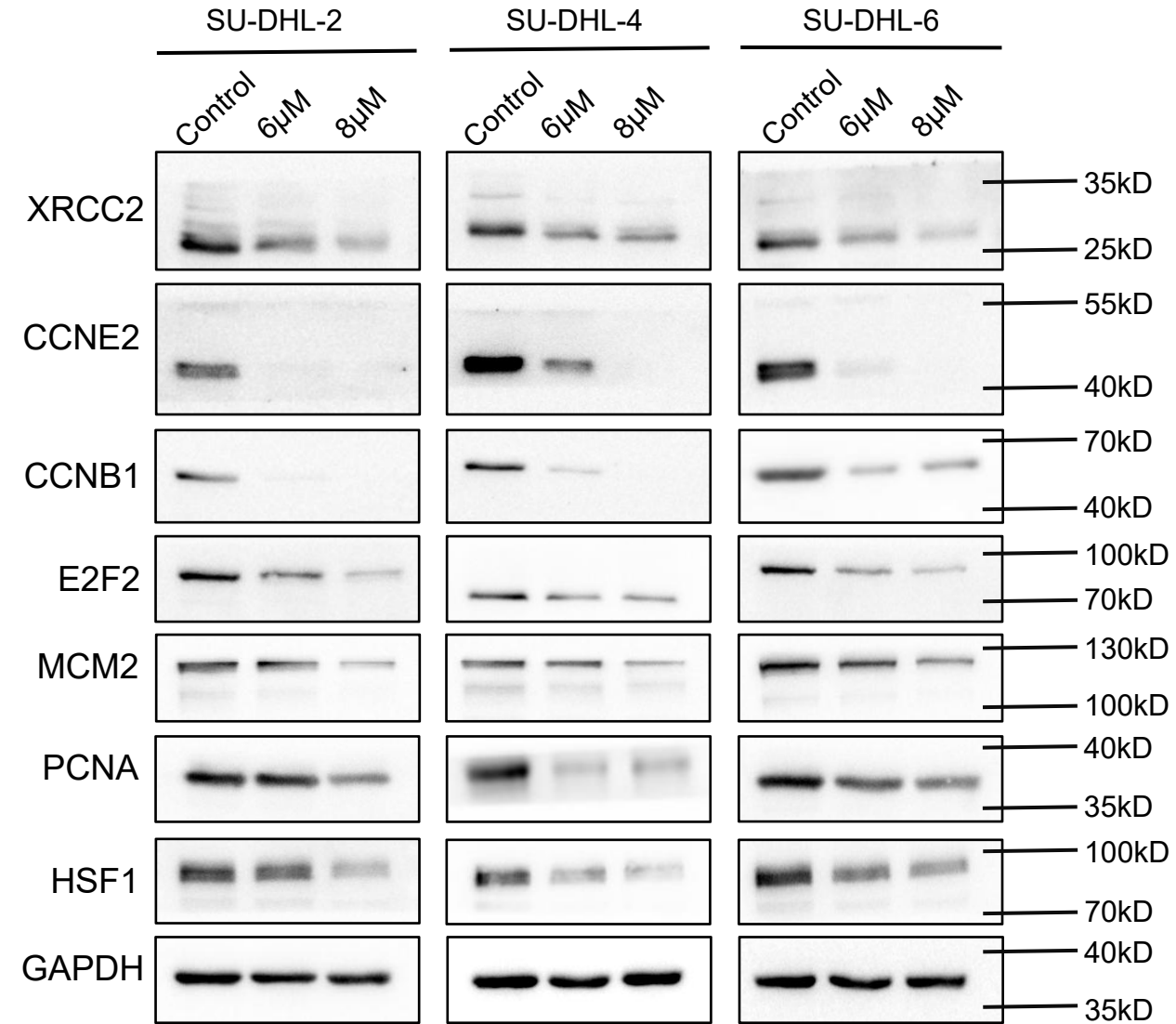

Figure S3

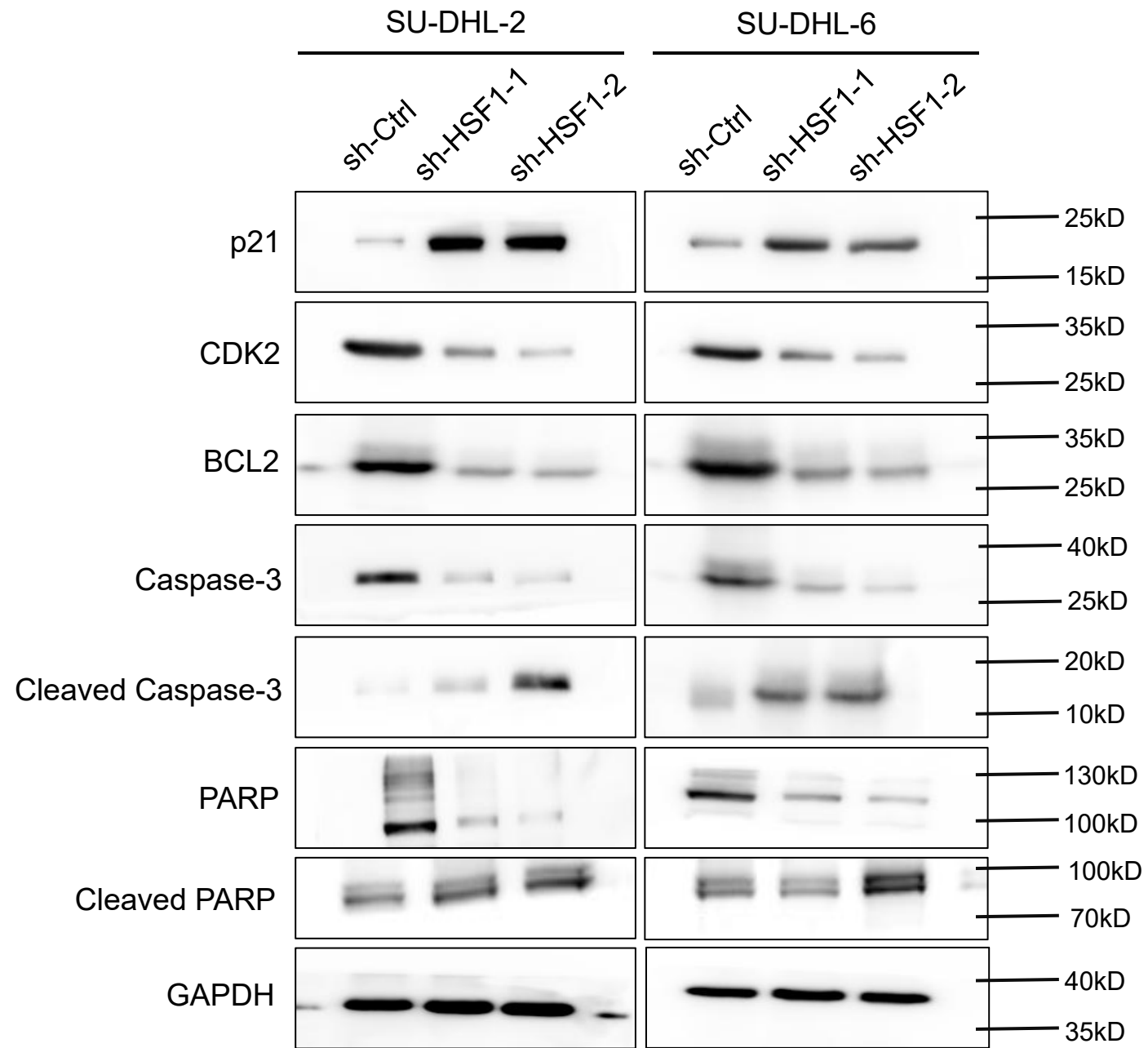

## Figure S5

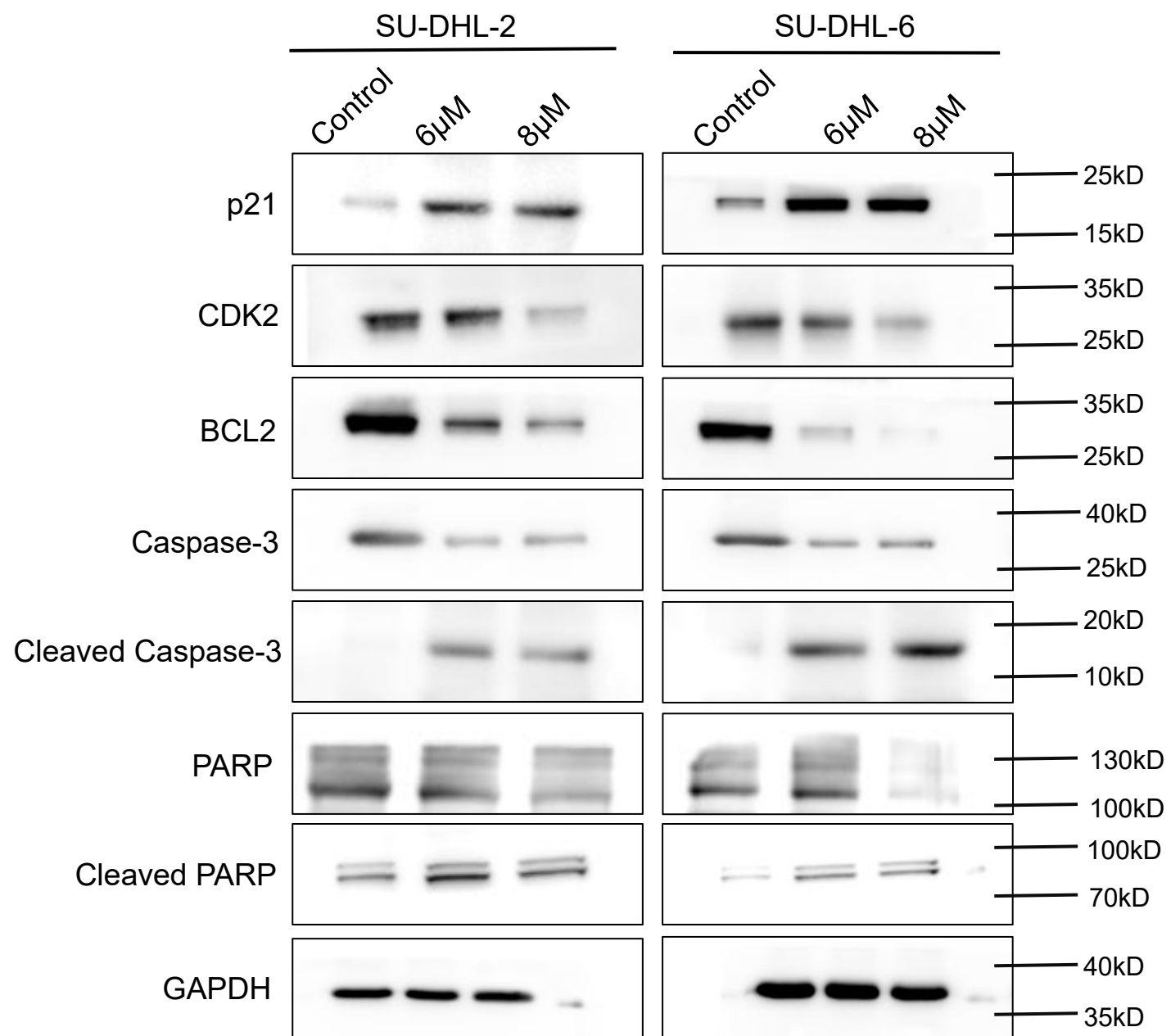

## Figure S8

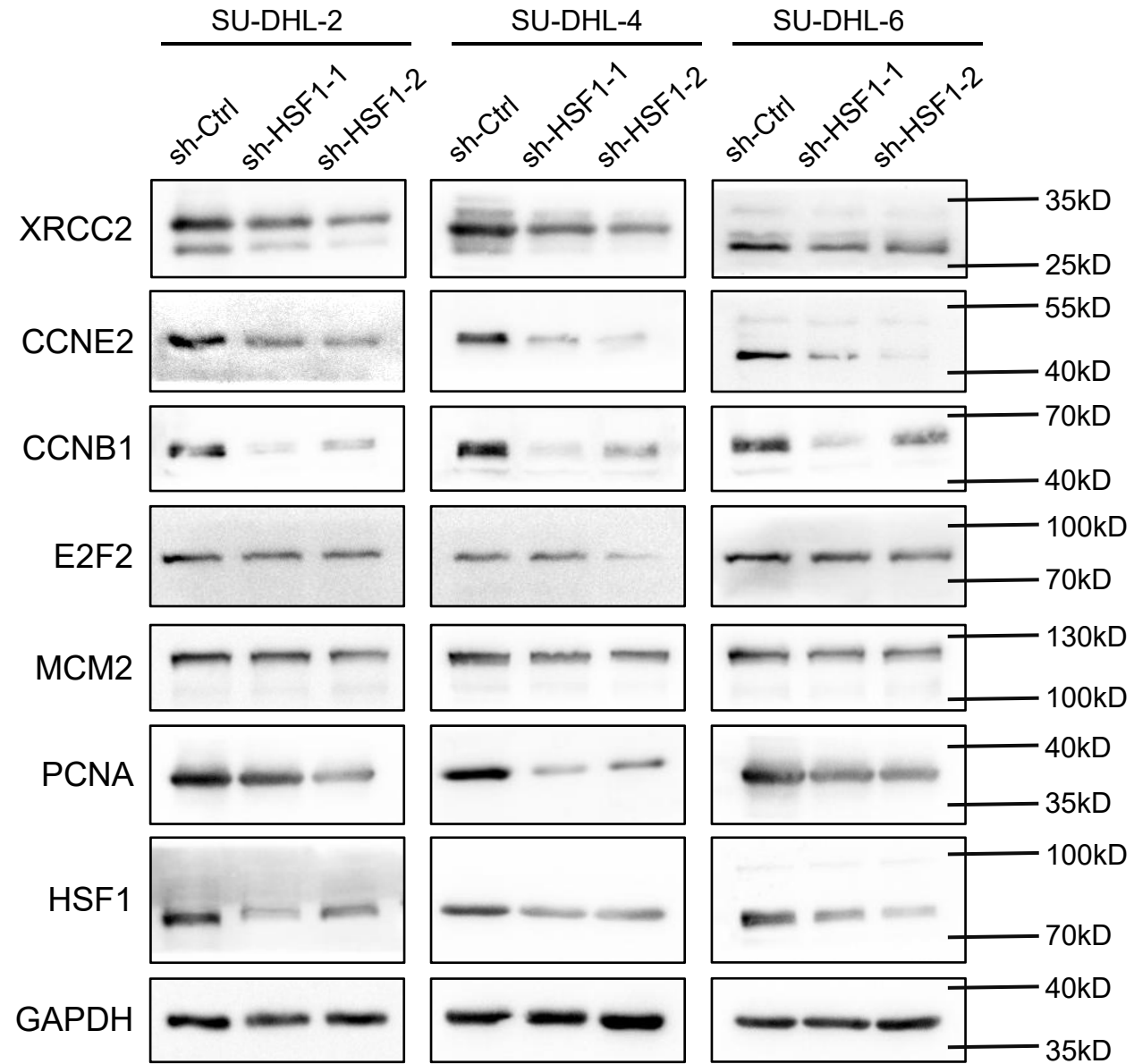

Supplement: Supplementary file 3 — Original western blots [file 41419_2025_7843_MOESM3_ESM.pdf]
